# Low-temperature fabrication of BTO-based relaxor ferroelectric thick films with multi-layered architecture

**DOI:** 10.1039/d6ra02102f

**Published:** 2026-05-18

**Authors:** Hongwei Zhang, Weibing Ma, Zhuo Zhang, Naihe Yi, JingNan Hong

**Affiliations:** a Key Laboratory of Advanced Ceramics and Machining Technology of Ministry of Education, Tianjin University Tianjin 300072 China maweibing@tju.edu.cn

## Abstract

Screen-printed BTO-based ceramic films conventionally suffer from issues such as high sintering temperatures incompatible with Ag electrodes, poor temperature stability, high porosity, and inferior electrical properties. In this study, the 0.3(Na_0.5_Bi_0.5_)TiO_3_-0.7(Ba_0.9_Sr_0.1_)(Ti_0.8_Zr_0.2_)O_3_ (0.3NBT-0.1Sr-0.2Zr) ferroelectric ceramic films were fabricated on alumina substrates with Ag electrodes *via* screen printing, followed by sintering at a maximum temperature of 900 °C. The effects of the lead-free glass additive and “sandwich” structure on the microstructure, dielectric properties, and energy storage properties of the ceramic films were systematically investigated. The optimized electrical properties of the multilayer ceramic thick film were achieved as follows: a dielectric constant of 585, a dielectric loss of 0.033, a temperature coefficient of dielectric constant of −39 ppm °C^−1^ (20–120 °C), a breakdown strength of 405 kV cm^−1^, an energy storage density of 1.65 J cm^−3^, a remanent polarization of 2.34 µC cm^−2^, and an energy storage efficiency of 74.3%. Low-temperature sintering of BaTiO_3_-based relaxor ferroelectric ceramic films with high energy storage properties was successfully achieved *via* screen printing.

## Abstract 2

A screen‑printed BTO-based thick film with lead-free glass and sandwich structure enables low-temperature sintering (900 ℃). It achieves a high energy density of 1.65 J cm^−3^, efficiency of 74.3%.

## Graphical abstract

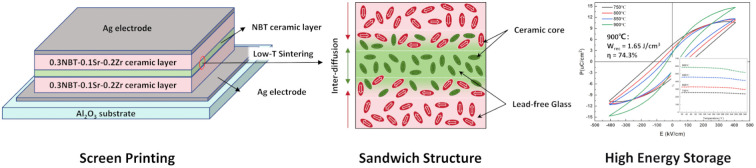

## 1 Introduction

Ferroelectric ceramic films have garnered tremendous attention in advanced energy storage systems, pulse power devices, and electronic components due to their superior dielectric properties, high polarization response, and excellent thermal stability.^1–3^ Among various ferroelectric materials, barium titanate (BTO)-based ceramics are regarded as promising candidates for energy storage applications, benefiting from their intrinsic high dielectric constant and adjustable ferroelectric behavior.^4^ However, several critical challenges hinder the practical application of this BTO-based system. Barium titanate ceramics typically exhibit an abrupt change in dielectric constant near the Curie temperature, which severely impairs the operational stability of electronic devices in wide-temperature-range applications and causes unpredictable performance fluctuations. Meanwhile, according to electrostatics, the overall energy storage density (*W*_total_), recoverable energy storage density (*W*_rec_), and efficiency (*η*) of dielectric capacitors can be determined *via* the following equations:
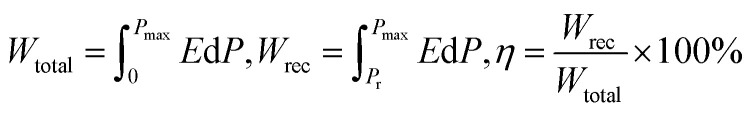
where *E*, *P*_max_, and *P*_r_ represent the applied electric field, the maximum polarization, and the remnant polarization, respectively. Consequently, high maximum polarization, high breakdown strength (*E*_b_), and minimal hysteresis are advantageous for achieving high *W*_rec_ and *η*. Relaxor ferroelectrics have superior potential and appear to be promising alternatives for energy storage applications because of their synergistically high saturated polarization (*P*_s_), low remnant polarization, high breakdown strength, and ultra-slim *P*–*E* loops.^5,6^

Compared with other film preparation techniques such as magnetron sputtering, the sol–gel method, spray coating method and tape-casting method, screen printing stands out as a cost-effective and scalable technique with simplified processing steps, making it more suitable for industrial mass production.^7^ However, when applied to BTO-based ceramic films, this method faces two key limitations. Firstly, screen-printed ceramic films typically suffer from poor densification due to the inherent limitations of the printing process, leading to compromised mechanical properties and electrical properties.^4^ Secondly, conventional BTO-based ceramics require high sintering temperatures, which not only poses compatibility challenges for low-melting-point electrodes (*e.g.*, Ag electrodes) and base metals—thereby increasing electrode costs—but also increases energy consumption. These factors collectively restrict the integration of such films into electronic devices.^8–10^

To address the aforementioned drawbacks inherent to BTO-based materials and screen-printing technology, researchers have proposed a series of optimization strategies.^11^ Ion doping and composite modification have been proven to effectively resolve the inherent performance defects of the pure BTO ceramics. For instance, Futakuchi *et al.*^12^ doped Zr^4+^ into Ba(Ti,Zr)O_3_ thick films, and Mahmoud *et al.*^13^ achieved Ca^2+^/Sn^4+^ co-doping in (Ba_1−*x*_Ca_*x*_)(Ti_0.9_Sn_0.1_)O_3_ ceramics, both regulating lattice distortion and phase transition to improve piezoelectricity and dielectric stability; He *et al.*^14^ developed BaTiO_3_-Bi(Mg_0.2_Ni_0.2_Zn_0.2_Zr_0.2_Nb_0.2_)O_3_ (BT-BMNZZN), leveraging synergistic effects between components to enhance maximum polarization, suppress dielectric loss, and induce strong relaxor behavior; Lv *et al.*^15^ designed BiFeO_3_-BaTiO_3_-SrTiO_3_ ternary composite systems, which further optimize performance *via* interface diffusion-induced solid solution formation. Low-temperature sintering additives and microstructure engineering have demonstrated significant potential in addressing the limitations of poor densification effect and high sintering temperature in screen printing. Researchers have employed various low-melting-point sintering aids (such as Li_2_O,^4^ Li_2_SiO_3_^16^, ZnBO,^8,9^ and ZnO/CuO^17^) to successfully reduce the sintering temperature of screen-printed BTO-based thick films to below 1000 °C, while enhancing their densification through liquid-phase sintering or grain refinement mechanisms.^8^ Zhao *et al.*^17^ developed a BT-BMZ@SiO_2_ “core–shell” structure in which insulating shells isolate highly polar clusters, while Li *et al.*^6^ designed a polar-slush/nonpolar cubic matrix that partitions polar regions. Both approaches effectively suppress polarization relaxation loss through cluster isolation, enhance breakdown strength, and hinder breakdown path propagation.^1,9^ Additionally, multilayered structures have been implemented to optimize overall performance. For example, Lv *et al.*^15^ developed alternating BF-ST/BST layered films (*N* = 10 cycles), and Yan *et al.*^18^ constructed BNKSTT/BNT-SNA “sandwich” structured ceramics. They leveraged the complementary properties and mutual diffusion of constituent layers to form a solid solution, thereby improving energy storage density and temperature stability.

In the field of low-temperature sintering of ferroelectric ceramic films, researchers have explored various approaches. Sadl *et al.*^19^ and Merselmiz *et al.*^20^ successfully prepared ferroelectric ceramic films on stainless steel substrates by aerosol deposition, with sintering temperatures as low as 500–800 °C. Yuan *et al.*^2^ introduced a cold sintering process into the screen-printing process, reducing the sintering temperature to 950 °C. Zhang *et al.*^21^ lowered the sintering temperature of screen-printed films (with Pt electrodes) to 950 °C by adding Bi-Li sintering aids. Despite these advances, most studies still require sintering temperatures above 950 °C, and the preparation processes are complex and costly. Few studies have successfully integrated low-temperature sintering and structural optimization design into the screen printing process to simultaneously achieve low-cost fabrication, low-temperature sintering, and synergistic optimization of dielectric and energy storage properties. Low-melting glass is an effective sintering aid and has been widely used for the low-temperature sintering of ceramics. Its incorporation not only reduces the sintering temperature, but also contributes to grain refinement, enhanced densification, and improved interfacial bonding. Moreover, it inhibits the volatilization of active components and forms an amorphous-nanocrystalline matrix that isolates polar clusters. This mechanism not only preserves high polarization intensity but also effectively improves the breakdown strength.^22^ PbO-containing glasses are popular glass additives owing to their desirable application properties.^23,24^ However, PbO is toxic to humans and animals, and lead-containing electronic waste can cause severe environmental damage.^11,25^ This has driven the development of PbO-free glasses for applications in the microelectronics industry. Alkali oxides and alkaline earth oxides increase the dielectric loss of the glass, which is undesirable for energy storage ceramics.^26^ Considering these drawbacks, a survey of low-softening point glasses would focus on glasses free from the oxides mentioned above.

In this work, a BTO-based composite system was selected, where doping with Sr^2+^ and Zr^4+^ ions enables “peak shifting” effects, while incorporating (Na_0.5_Bi_0.5_)TiO_3_ (NBT) induces relaxor ferroelectric characteristics. These modifications result in the formation of 0.3[(Na_0.5_Bi_0.5_)TiO_3_]-0.7[(Ba_0.9_Sr_0.1_)(Ti_0.8_Zr_0.2_)O_3_] (abbreviated as 0.3NBT-0.1Sr-0.2Zr), a composite system that exhibits prominent advantages such as a high dielectric constant, low dielectric loss at room temperature, and broad temperature stability. An environmentally friendly lead-free ZnO-B_2_O_3_-Bi_2_O_3_ glass additive was synthesized, which avoids the hazards caused by lead-containing glass additives, while featuring a low melting point, high resistivity, and excellent fluidity.^27–30^ To further develop a low-temperature-sintered ferroelectric ceramic film with high dielectric properties, high breakdown strength, and favorable energy storage properties, a “sandwich” structure with the configuration 0.3NBT-0.1Sr-0.2Zr/NBT/0.3NBT-0.1Sr-0.2Zr was designed, leveraging the complementary ferroelectric and energy storage properties between NBT (excellent relaxor behavior and low remanent polarization) and BTO (high maximum polarization and dielectric constant).^18,31^ The multi-layered architecture promotes interfacial diffusion to form solid solutions, further enhances relaxor characteristics, and hinders the propagation of breakdown paths—thereby collectively optimizing the energy storage properties.^16,32^

## 2 Materials and methods

### 2.1 Preparation of thick films

0.3[(Na_0.5_Bi_0.5_)TiO_3_]-0.7[(Ba_0.9_Sr_0.1_)(Ti_0.8_Zr_0.2_)O_3_] (abbreviated as 0.3NBT-0.1Sr-0.2Zr) powder was prepared through a traditional solid state method. The starting materials, including BaCO_3_ (99.7%), SrCO_3_ (99.7%), TiO_2_ (99.7%), ZrO_2_ (99.7%), Bi_2_O_3_ (99.7%), Na_2_CO_3_ (99.7%), were weighed according to the molar ratio and subjected to ball milling with agate balls for 4 h in water. The resulting dried powders were calcined at 850 °C for 1 h, and then calcined at 1150 °C for 3 h. (Na_0.5_Bi_0.5_)TiO_3_ were also prepared through a traditional solid state method. Bi_2_O_3_ (99.7%), Na_2_CO_3_ (99.7%), TiO_2_ (99.7%), were weighed according to a Bi/Na/Ti molar ratio = 1 : 1 : 2. They were subjected to ball milling with agate balls for 4 h. The resulting dried powders were pre-sintered at 850 °C for 1 h, and then calcined at 1150 °C for 3 h. ZnO-B_2_O_3_-Bi_2_O_3_ glass burning aid was prepared through a water quenching method. The starting materials, including ZnO (99.7%), B_2_O_3_ (99.7%), Bi_2_O_3_ (99.7%), were mixed according to a Zn/B/Bi molar ratio = 1 : 1 : 8. They were melted at 1200 °C for 1 h. The molten material was taken out and poured into cold water for quenching to form glass powder. All the synthesized powders were further milled using ZrO_2_ balls for 8 h to achieve a fine particle size and dried for later use. 0.3[(Na_0.5_Bi_0.5_)TiO_3_]-0.7[(Ba_0.9_Sr_0.1_)(Ti_0.8_Zr_0.2_)O_3_] (0.3NBT-0.1Sr-0.2Zr) ceramic powder and glass powder were mixed according to a mass percentage ratio (*e.g.*, the glass powder accounting for 0 wt%, 3 wt%, 5 wt%, 7 wt%, and 9 wt% of the total powder mass).

The preparation process of the screen-printing slurry is described in the following. The paste of the screen-printing slurry was prepared with terpineol, ethyl cellulose and titanate coupling agent as raw materials. Their addition ratios and functions are shown in Table 1. They were mixed and stirred in a water bath at 70 °C until the ethyl cellulose was completely dissolved, then removed and cooled to room temperature. The pre-mixed powder was sequentially added to the solution at a mass ratio of 3 : 1, and the mixtures were stirred at 70 °C for 1 h to ensure the homogeneous dispersion of the powders in the solution.

**Table 1:** Composition of the screen printing paste solution

Component	Mass ratio (wt%)	Function
Terpineol	85	Solvent
Ethyl cellulose	10	Binder
Titanate coupling agent	5	Dispersant

Thick film was fabricated by the screen-printing technique. Alumina substrates with dimensions of 25 × 20 × 1 mm and 300-mesh silk screens were employed in the experiments. Ag paste with a printed area of 20 × 18 mm was printed on the substrates followed by sintering at 850 °C for 30 min to prepare the bottom electrode. Then, ∼22 µm-thick 0.3NBT-0.1Sr-0.2Zr films with a size of 15 × 15 mm were prepared on the bottom electrodes. After each printing, the films were dried in an oven at 120 °C and then cooled to room temperature before the next printing. This process was repeated three times to obtain the green ceramic thick film. A high isostatic pressure of 200 MPa was applied on the green films before sintering. The films were heated at a rate of 1 °C min^−1^ and held at 150 °C, 250 °C, and 350 °C for 30 min at each temperature to remove organic binders from the green films. Afterwards, the films were heated at a heating rate of 5 °C min^−1^, held at 620 °C for 1 h, and further annealed at the maximum sintering temperatures (750/800/850/900 °C) for 3 h. The top electrode, also a screen-printed Ag paste with a diameter of 10 mm, was calcined at 720 °C for 60 min.

When printing the thick films, a “sandwich” structured multilayer was adopted in place of the single-layer thick film. The first layer of 0.3NBT-0.1Sr-0.2Zr thick film was screen-printed on the bottom electrode. After each printing, the films were also dried in an oven at 120 °C and then cooled to room temperature. This process was repeated two times. Then NBT thick film (same mass fraction of glass as 0.3NBT-0.1Sr-0.2Zr thick film) was prepared on the first layer, and only printed once as the interlayer. Finally, the 0.3NBT-0.1Sr-0.2Zr thick film, identical to the first layer, was screen-printed twice on the NBT layer to complete the preparation of the sandwich structure ceramic film. The isostatic pressure and sintering schedule as above were repeated. After sintering, the thickness of the single 0.3NBT-0.1Sr-0.2Zr layer was measured to be ∼16 µm, the NBT layer ∼7 µm, and the multilayer total ∼40 µm.

### 2.2 Characterization and testing methods

The phase of the fabricated thick films was characterized using the X-ray diffraction (XRD- Brüker D8 Advance diffractometer equipped with a copper anode emitting, a Cu Kα radiation source (*λ*_Cu Kα = 1.5406 Å)) with a scanning rate of 0.01 per step between 2*θ* = 20° and 80°. The microstructure characteristics, thickness, and element distributions of ceramic films were characterized *via* a field emission scanning electron microscopy (FE-SEM; S-4800, Hitachi Limited, Japan). The porosity of the sintered thick films was quantitatively evaluated from the SEM images using ImageJ software. For each sample, three representative SEM images were taken at different magnification. A suitable threshold was applied to distinguish pores from the solid ceramic phase. The pore area fraction was then automatically calculated by the software, and the average value from the multiple images was taken as the overall porosity of the sample.

The polarization–electric field (*P*–*E*) curves were carried out by a ferroelectric tester (TF Analyzer 2000E, AixACCT, Germany) at 10 Hz. For each group of three identical samples, the average withstand electric field strength was taken as the breakdown strength. The *P*–*E* loops at that field strength were measured for that sample. The recoverable energy storage density and energy storage efficiency were calculated by integrating the *P*–*E* loops using Origin software. The overall energy storage density (*W*_total_) was obtained as the integral of the polarization with respect to the electric field from zero to the maximum polarization. The energy storage density (*W*_rec_) was determined by integrating the area between the polarization curve and the polarization axis from the remanent polarization (*E* = 0) to the maximum polarization. The efficiency (*η*) of dielectric capacitors was then calculated as *η* = *W*_rec_/*W*_total_ × 100%. For each sample, at least three *P*–*E* loops were integrated, and the average values were reported. The capacitance (*C*) was tested on an LCR automatic meter (TH2810B) at 1 MHz.1
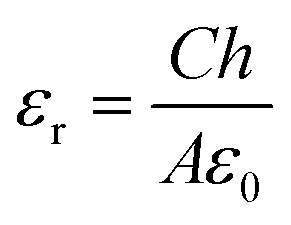
where *ε*_r_ is the dielectric constant of the ceramic films, *h* is the thickness of the ceramic films, *ε*_0_ is the vacuum dielectric constant, and *A* is the electrode area.2
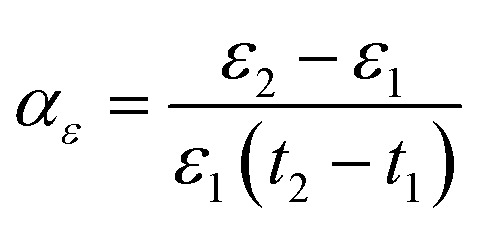
where *α*_*ε*_ represent the temperature coefficient of dielectric constant of the ceramic films, *ε*_1_, and *ε*_2_, respectively represent the dielectric constants at the temperature *t*_1_= 20 °C and *t*_2_= 120 °C.

## 3 Results and discussions

### 3.1 Crystal composition analysis

**Fig. 1:**
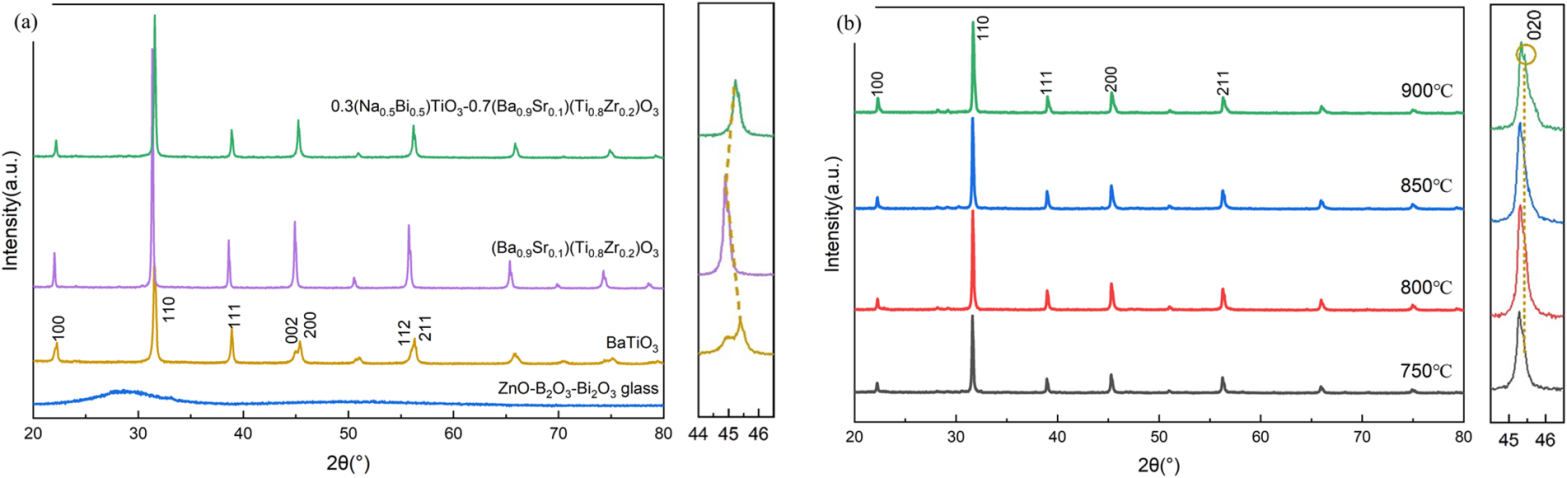
(a) XRD pattern of the as-synthesized powder and local enlarged view near 45°, (b) XRD pattern of 0.3NBT-0.1Sr-0.2Zr ceramic thick film sintered at different temperatures and local enlarged view near 45°.

Fig. 1(a) show the XRD pattern (2*θ* range of 20–80°) of the ZnO-B_2_O_3_-Bi_2_O_3_ glass powder exhibits only a broad amorphous halo without any crystalline peaks. Meanwhile, the XRD pattern of BaTiO_3_, (Na_0.5_Bi_0.5_)TiO_3_, (Ba_0.9_Sr_0.1_)(Ti_0.8_Zr_0.2_)O_3_, and 0.3(Na_0.5_Bi_0.5_)TiO_3_-0.7(Ba_0.9_Sr_0.1_)(Ti_0.8_Zr_0.2_)O_3_ ceramic powder, along with their corresponding local magnified views near 45°. All the diffraction peaks observed for the 0.3(Na_0.5_Bi_0.5_)TiO_3_-0.7(Ba_0.9_Sr_0.1_)(Ti_0.8_Zr_0.2_)O_3_ powder match well with those of perovskite phase. After doping and compositing, the crystal structure transformed from tetragonal BTO to a pseudo-cubic phase, and no impurity peaks were detected. After co-doping with Sr^2+^ and Zr^4+^, the lattice constant increases due to the stronger influence of Zr^4+^. Conversely, the incorporation of NBT reduces the lattice constant. The (002)/(200) peaks merge into a unimodal pattern (200) peak. The (200) diffraction peak first shifts toward lower angles and then moves back to higher angles, confirming the successful preparation of NBT-composited and Sr/Zr co-doped BTO-based ceramic powder.

Fig. 1(b) shows the XRD patterns of 0.3NBT-0.1Sr-0.2Zr thick films sintered at different temperatures, along with their corresponding local magnified views near 45°. Regardless of the sintering temperature, all samples mainly exhibited the pseudo-cubic phase. In addition, small secondary peaks gradually appeared near 30° and 45° as the sintering temperature increased. This was attributed to the compositing of the NBT phase. As shown in Fig. 1(b), the (020) peak of the rhombohedral phase near 45° became progressively sharper. The higher sintering temperature provided stronger driving force for sintering. This facilitated better integration of NBT, disrupting the original long-range ordered structure. This process would effectively improve the relaxor characteristics and enhanced the electrical properties.

### 3.2 Effect of glass content on the microstructure and electrical properties of ceramic thick films

**Fig. 2:**
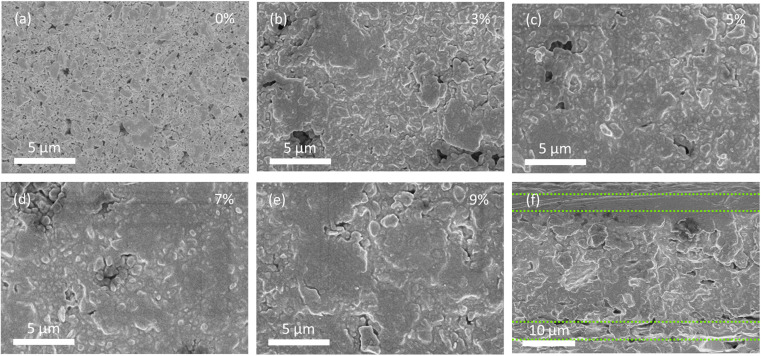
(a–e) SEM images of 0.3NBT-0.1Sr-0.2Zr ceramic films sintered at 800 °C with different glass mass fractions: (a) 0 wt%, (b) 3 wt%, (c) 5 wt%, (d) 7 wt%, (e) 9 wt%, (f) cross-sectional SEM image of the ceramic film.

The low-melting glass additive melts into a liquid phase during sintering, reducing the activation energy required for ceramic particle diffusion and promoting particle rearrangement, thereby achieving higher densification and a lower sintering temperature. Fig. 2(a–e) respectively show SEM images of the 0.3NBT-0.1Sr-0.2Zr ceramic thick films with glass powder mass fractions of 0 wt%, 3 wt%, 5 wt%, 7 wt%, and 9 wt% in the total ceramic powder, all sintered at 800 °C. Fig. 2(f) shows the thick film cross-section, and the green line represent the top and bottom electrodes. The thick film thickness was ∼22 µm. Without glass addition (0 wt%), the sintering temperature was insufficient, resulting in small ceramic particles and high porosity. The average pore size was ∼0.27 µm. The porosity calculated by ImageJ was ∼7.43%. At 3 wt% glass content, sintering occurred between ceramic particles and the porosity decreased significantly to 2.31%. However, the low glass proportion was still inadequate to completely fill the defects, leaving some pores or even formed large voids. The maximum size reached 1.65 µm. These large pores acted as weak points for electrical breakdown, severely impairing the dielectric loss and the breakdown strength. When the glass content increased to 5 wt%, the thick film became relatively dense, with a porosity of 0.52% and a maximum pore size reduced to 0.86 µm. At 7 wt%, the porosity decreased to 0.34%. Most pores were filled by the glass phase, which would improve the electrical properties. At 9 wt%, the porosity was as low as 0.24% and the ceramic was fully dense. However, the surface was covered by an excessive liquid phase, which would degrade the dielectric response and the polarization strength.

**Fig. 3:**
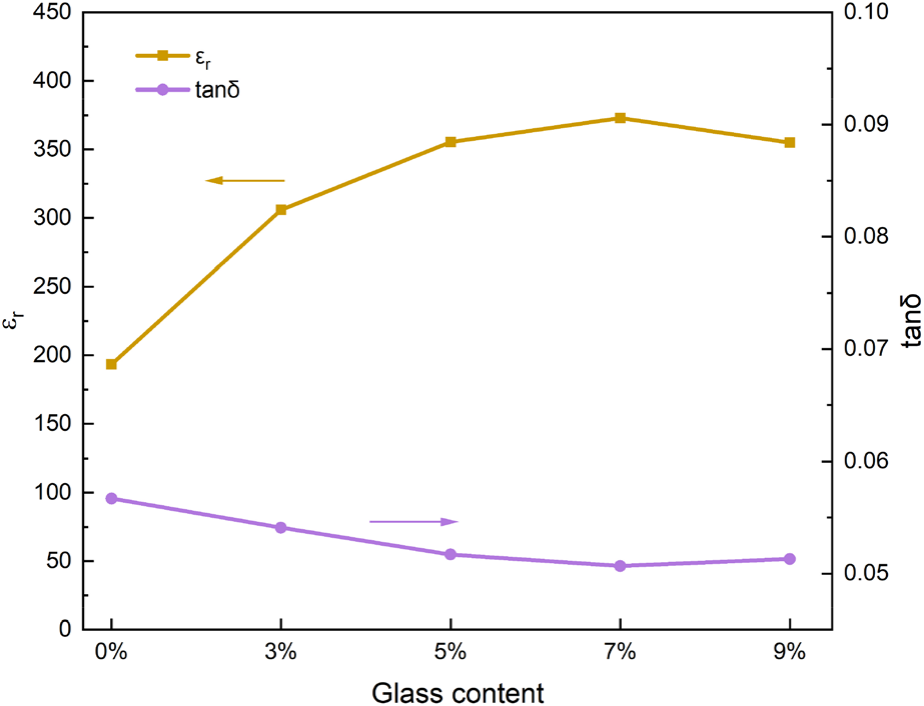
Dielectric properties curves of 0.3NBT-0.1Sr-0.2Zr ceramic films (sintered at 800 °C) with different glass mass fractions.

Fig. 3 shows the dielectric properties of 0.3NBT-0.1Sr-0.2Zr ceramic thick films with different glass contents (sintered at 800 °C). As shown, the glass powder significantly affected the electrical properties of the films. At 0 wt% glass, the dielectric constant was only 193 and the dielectric loss was 0.057. These poor electrical properties resulted from the insufficient sintering temperature, inadequate grain growth, and high porosity. With 3 wt% glass, the glass acted as a liquid phase that lowered the sintering temperature and filled pores, so the dielectric constant increased noticeably to 305. As the glass content increased further, the pores were gradually filled. This led to improved densification and a significant reduction in defects. Consequently, the dielectric constant increased gradually and the dielectric loss decreased. At 7 wt% glass, the ceramic thick film became nearly dense, achieving a dielectric constant of 372 and a dielectric loss of 0.050. Beyond this point, further increasing the glass fraction did not continue to significantly reduce the porosity. The reduced proportion of ceramic led to a decrease in dielectric constant. Overall, the dielectric properties first increased and then decreased with rising glass content, reaching a maximum at 7 wt% glass.

**Fig. 4:**
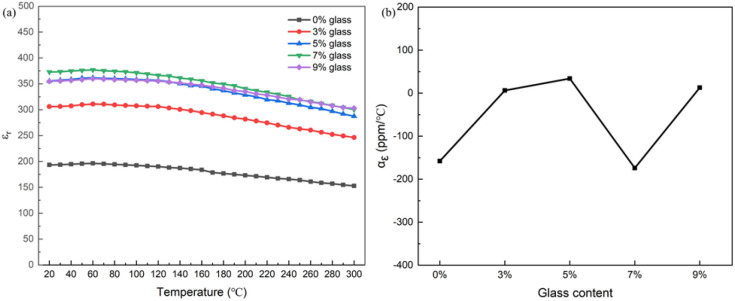
(a) Temperature-dependent dielectric constant curves (20–300 °C), (b) temperature coefficient of dielectric constant (20–120 °C) of 0.3NBT-0.1Sr-0.2Zr ceramic thick films (sintered at 800 °C) with different glass contents.

Fig. 4 shows the temperature-dependent dielectric constant and corresponding temperature coefficients of 0.3NBT-0.1Sr-0.2Zr ceramic thick films (sintered at 800 °C) with different glass contents. All samples exhibited typical relaxor ferroelectric characteristics across the entire test temperature range. At 0 wt% glass content, the dielectric constant was relatively low, with a temperature coefficient of dielectric constant of −158 ppm °C^−1^ (20–120 °C). As the glass content increased, the dielectric constant gradually rose, reaching its maximum at 7 wt%, where the temperature coefficient was −174 ppm °C^−1^. With a further increase to 9 wt%, the dielectric constant decreased, and the temperature coefficient was 13 ppm °C^−1^. Throughout all compositions, the temperature coefficient of dielectric constant remained at a low level, indicating good dielectric stability. Notably, the increase in glass content markedly improved the dielectric stability of the thick films in the high-temperature region (>120 °C). The dielectric constant curves at high temperatures became flatter. As the glass phase gradually increased, the porosity decreased. The influence of defects on the dielectric response was weakened.^33^ The dense structure restricted excessive thermal disturbance of polar nanoregions (PNRs) at elevated temperatures, delaying the decay of polarization ordering^34^

**Fig. 5:**
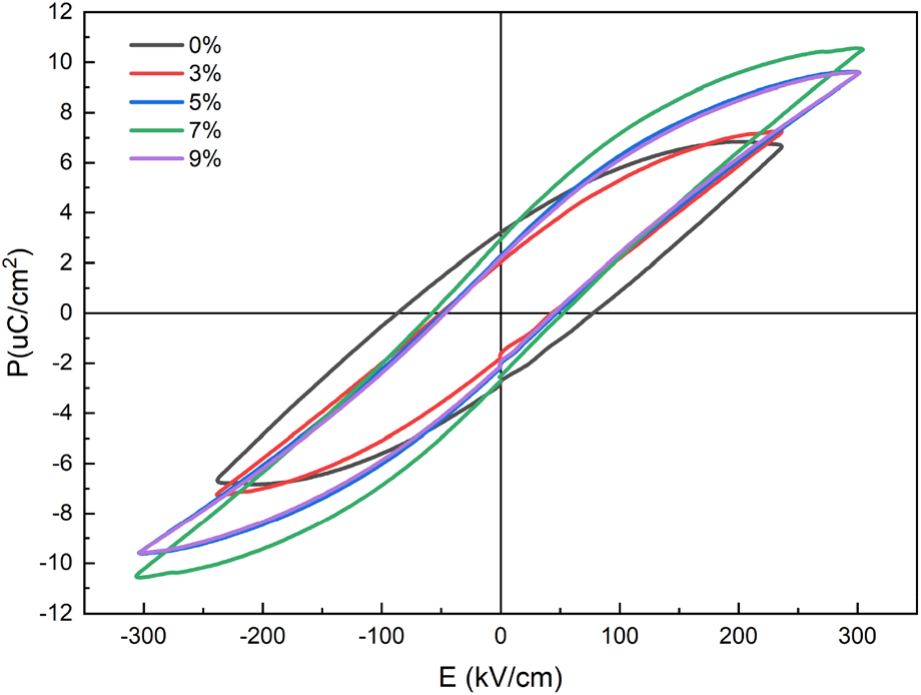
*P*–*E* loops of 0.3NBT-0.1Sr-0.2Zr ceramic thick films (sintered at 800 °C) with different glass mass fractions.

As the porosity decreases, the influence of defects on the polarization response is weakened, resulting in a reduction of remanent polarization. In addition, the dense structure hinders the propagation of breakdown paths, leading to an increased breakdown strength. Fig. 5 shows the *P*–*E* loops of 0.3NBT-0.1Sr-0.2Zr ceramic thick films with different glass contents. Without glass addition (0 wt%), the *P*–*E* loop was flat, exhibiting high remanent polarization and coercive field. This was attributed to the numerous internal defects in the ceramic film and its weak ferroelectricity, leading to non-uniform internal electric fields and high leakage current. At 3 wt% glass, pores were partially filled. This significantly reduced the friction loss between ferroelectric domains, thereby decreasing both remanent polarization and coercive field.^35^ However, due to the growth of ceramic grains and insufficient glass addition, some large pores remained, resulting in a low breakdown strength. As the glass content increased, the porosity of films decreased and ferroelectricity of the ceramic was enhanced. This enhanced breakdown strength, and the polarization strength rose. At 7 wt% glass, the breakdown strength reached 305 kV cm^−1^, energy storage density was 0.78 J cm^−3^, remanent polarization was 2.93 µC cm^−2^, and energy storage efficiency was 58.0%. When the glass content further increased to 9 wt%, the proportion of ferroelectric domains decreased, leading to a drop in polarization strength. Thus, polarization strength first increased and then decreased, peaking at 7 wt% glass, a trend consistent with the variation in dielectric constant.

### 3.3 Effect of sintering temperature on the microstructure and electrical properties of ceramic thick films

**Fig. 6:**
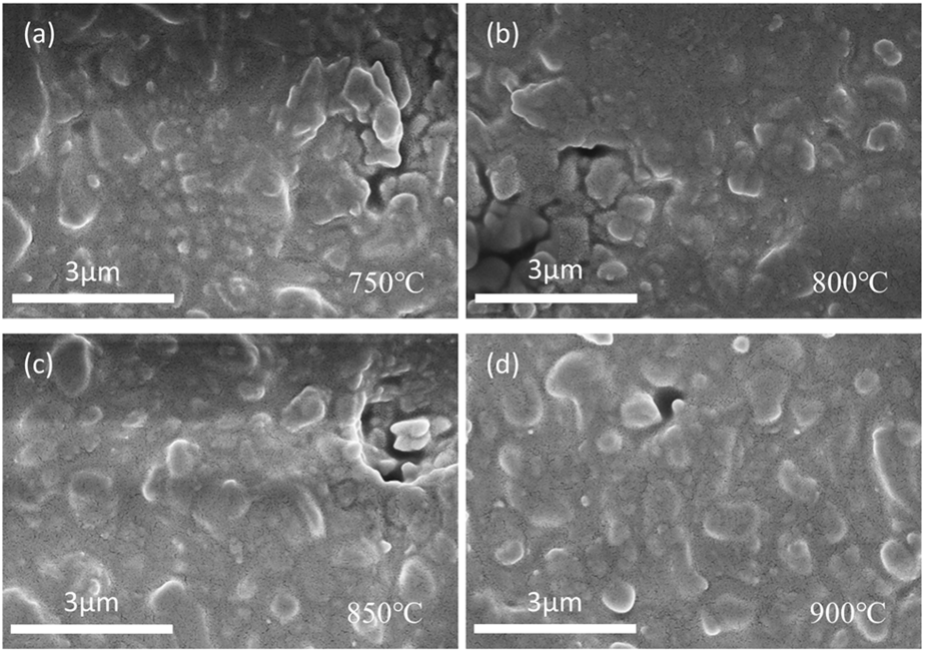
SEM images of 0.3NBT-0.1Sr-0.2Zr thick film with 7 wt% glass sintered at (a) 750 °C, (b) 800 °C, (c) 850 °C, (d) 900 °C.

Elevated temperature acts as the key driving force for the sintering densification process. Increasing the sintering temperature improves the fluidity of the molten glass. It also promotes better rearrangement between the liquid glass phase and ceramic particles. Fig. 6(a–d) shows SEM images of 0.3NBT-0.1Sr-0.2Zr thick films with 7 wt% ZnO-B_2_O_3_-Bi_2_O_3_ glass powder sintered at different temperatures. The densification of the ceramic did not change significantly with increasing sintering temperature, but the ceramic particles were uniformly dispersed by the glass phase. At the sintering temperature of 750 °C, the molten glass filled the ceramic interparticle voids reasonably well, but distinct agglomeration of ceramic particles was still observable (Fig. 6(a)). The porosity was 0.37%. There were numerous interconnected pores between ceramic particles with clearly defined particle boundaries. With increasing sintering temperature, ceramic particles became more dispersed by the liquid phase, and density increased (Fig. 6(b and c)). At 850 °C, the porosity decreased to 0.15%. As a result, the liquid phase filled the interparticle pores more uniformly. At 900 °C, most pores were completely filled, and the liquid phase uniformly encapsulated the ceramic grains, forming a continuous and dense structure.

**Fig. 7:**
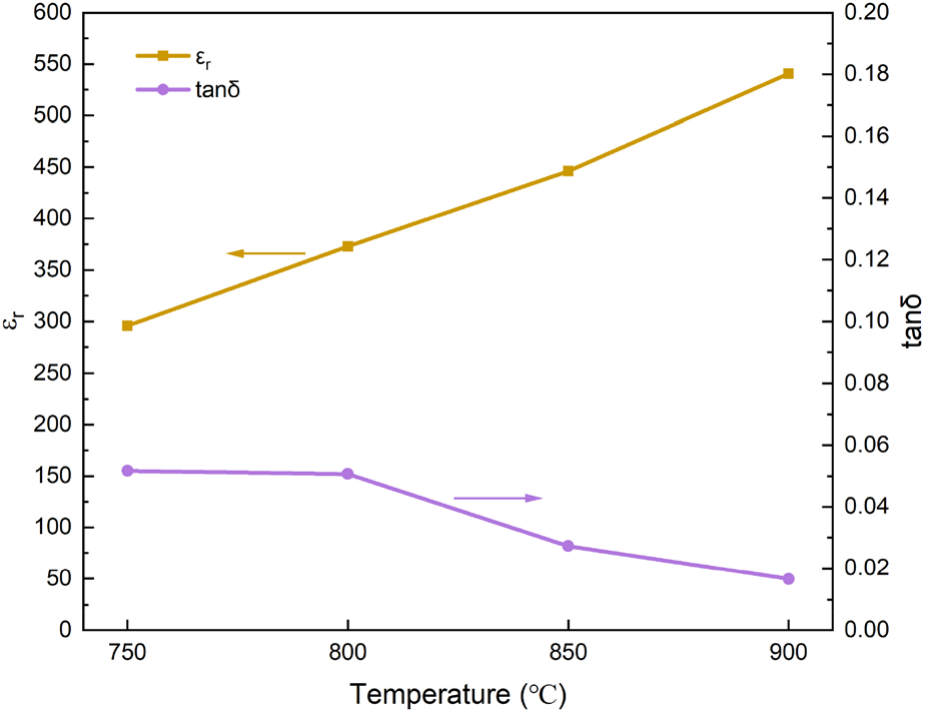
Dielectric properties curves of 0.3NBT-0.1Sr-0.2Zr thick film with 7 wt% glass sintered at different temperatures.

Fig. 7 shows the dielectric properties of 0.3NBT-0.1Sr-0.2Zr thick film with 7 wt% glass sintered at different temperatures. At the sintering temperature of 750 °C, the film exhibited a dielectric constant of only 295 and a dielectric loss of about 0.052. A lower sintering temperature resulted in weak ferroelectricity of the ceramic and inhomogeneous distribution between the liquid phase and the ceramic. Consequently, the dielectric response was weak and the frictional loss of domain walls was high. As the sintering temperature increased to 800 °C and 850 °C, the dielectric constant rose to 373 and 446, respectively, while the dielectric loss decreased to 0.050 and 0.027. As the sintering temperature increased, the ferroelectric domains grew better. Meanwhile, the enhanced fluidity of the glass phase effectively filled the interparticle pores, promoted particle rearrangement, and improved densification. This effectively suppressed leakage loss, space charge polarization loss, and reduced frictional loss.^36^ Thus, the dielectric properties were significantly improved. At 900 °C, the thick film exhibited optimal dielectric properties: the dielectric constant reached 541, the dielectric loss decreased to 0.016. This indicates that at this temperature, the ferroelectric phase was fully developed, and the glass fluidity was optimal. Therefore, it has the highest dielectric constant and the lowest dielectric loss at 900 °C.

**Fig. 8:**
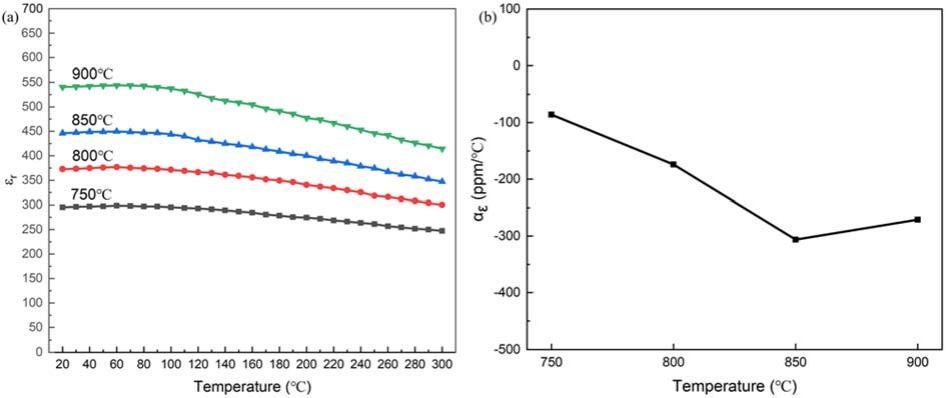
(a) Temperature-dependent dielectric constant curves (20–300 °C), (b) temperature coefficient of dielectric constant (20–120 °C) of 0.3NBT-0.1Sr-0.2Zr thick films with 7 wt% glass prepared at different sintering temperatures.

Fig. 8 shows the temperature-dependent dielectric constant and corresponding temperature coefficients of 0.3NBT-0.1Sr-0.2Zr thick films with 7 wt% glass prepared at different sintering temperatures. From the overall trend of the dielectric–temperature curves, all samples exhibited typical characteristics of relaxor ferroelectrics. At a sintering temperature of 750 °C, the dielectric constant was relatively low, with a temperature coefficient of dielectric constant of −86 ppm °C^−1^ (20–120 °C). As the sintering temperature increased, the dielectric constant gradually rose, and the temperature coefficient of dielectric constant also increased slightly. At a sintering temperature of 900 °C, the dielectric constant reached its maximum, with a temperature coefficient of −271 ppm °C^−1^. These were attributed to more complete grain growth and higher densification at elevated temperatures. They enhanced the dielectric response but also led to a slightly increased sensitivity of the material to temperature variations. Overall, the temperature coefficient of dielectric constant remained at a low level throughout.

**Fig. 9:**
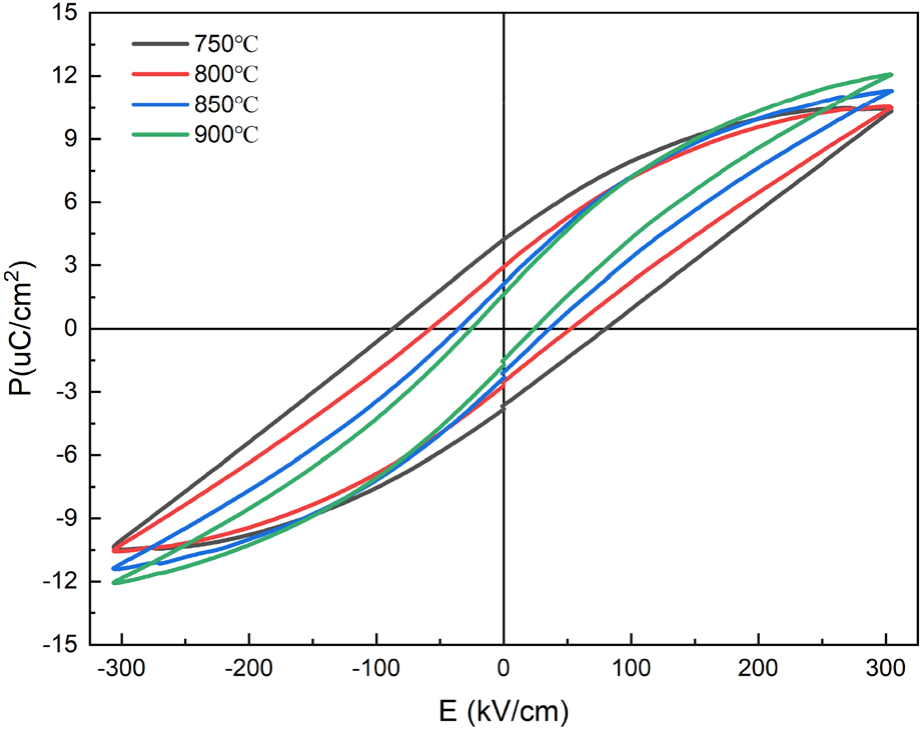
*P*–*E* loops of 0.3NBT-0.1Sr-0.2Zr thick films with 7 wt% glass prepared at different sintering temperatures.

Fig. 9 shows the energy storage properties of 0.3NBT-0.1Sr-0.2Zr thick films prepared at different sintering temperatures. It can be observed that the sintering temperature significantly influenced the ferroelectric and energy storage properties of the thick films. At the sintering temperature of 750 °C, ferroelectric domains were not fully developed, and the friction loss in the electric field was high. They led to a high remanent polarization and significant energy loss. The *P*–*E* loop exhibited a “fat” shape, with an energy storage density of only 0.58 J cm^−3^, a high remanent polarization of 4.22 µC cm^−2^, and an energy storage efficiency of ∼42.07%. As the sintering temperature increased to 800 °C and 850 °C, the *P*–*E* loops gradually became “slimmer”. On the one hand, with increasing temperature, the NBT phase continuously grew and composited the 0.3NBT-0.1Sr-0.2Zr phase, leading to progressively enhanced relaxor behavior. On the other hand, the liquid phase more uniformly coated the ceramic particles, and the high-resistivity glass shell effectively isolated electrical contact between highly polar grains. Consequently, remanent polarization was lowered, and the overall energy storage properties improved. At 900 °C, the film exhibited a breakdown strength of 305 kV cm^−1^, an energy storage density of 1.14 J cm^−3^, a remanent polarization of 1.64 µC cm^−2^, and an energy storage efficiency of 77.8%. This indicated the formation of a well-developed composite structure, and the insulating glass uniformly and completely encapsulated the highly polar BTO-based relaxor ferroelectric grains. Increasing the sintering temperature enhanced the fluidity of the glass phase and promoted the full development of the relaxor ferroelectric phase. This not only enhanced the breakdown strength and increased the energy storage density, but also reduced the frictional loss during polarization switching, thereby improving the energy storage efficiency. The energy storage properties of the thick films were systematically optimized.

**Fig. 10:**
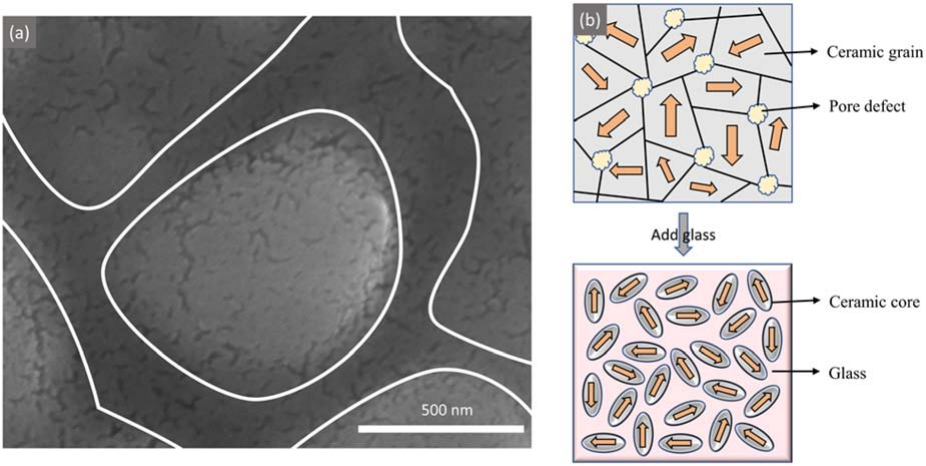
(a) SEM image of 0.3NBT-0.1Sr-0.2Zr ceramic with 7 wt% glass at sintering temperature of 900 °C; (b) schematic diagram of the ceramic structure before and after glass addition.

Fig. 10(a) shows the high magnification SEM image of 0.3NBT-0.1Sr-0.2Zr ceramic with 7 wt% glass at sintering temperature of 900 °C. The ZnO-B_2_O_3_-Bi_2_O_3_ glass exhibited high fluidity and good wettability with the ceramic. As a result, individual ceramic grains were clearly observed to be encapsulated by the glass phase, forming a “core–shell” structure. As shown in Fig. 10(b), the glass fills the pores and physically isolates adjacent grains, which improved densification and breakdown strength. In addition, this structure effectively restricted the thermal fluctuations of polar nanoregions (PNRs), improving the temperature stability. Meanwhile, it reduced the frictional loss of ferroelectric domains during switching under an electric field.^6,17^ The energy storage efficiency was enhanced. Wang *et al.*^37^ optimized the performance of Ba(Zr,Ti)O_3_ ceramic bulks through a sandwiched core@double-shell architecture, achieving a 40% increase in breakdown strength and a 59% increase in energy storage density.

### 3.4 Effect of “sandwich” multilayer structure on the microstructure and electrical properties of ceramic films

**Fig. 11:**
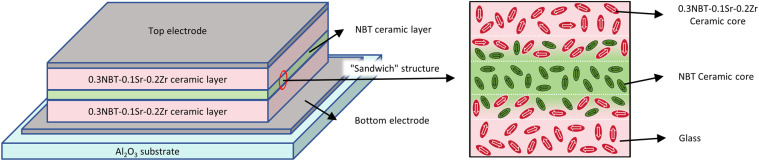
Schematic diagram of the sandwich-structured multilayer ceramic after sintering.

The glass additive eliminates interfacial stress between different ceramic layers and enhances interfacial diffusion. As shown in Fig. 11, a sandwich structure is designed. During sintering, different ceramic layers interdiffuse, forming a compositional gradient at the interfaces. This not only improves temperature stability but also hinders the propagation of breakdown paths. Finally, a ceramic thick film with excellent electrical properties is obtained.

**Fig. 12:**
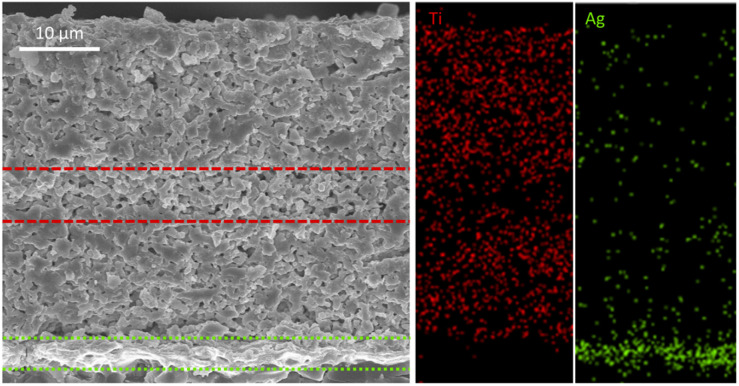
SEM and EDS mapping images of the multilayer ceramic thick film (7 wt% glass, sintered at 900 °C): Ti distribution (red) and Ag distribution (green).

NBT ceramic possesses high polarization strength and excellent relaxor characteristics,^38^ which can complement the properties of the BTO-based ceramic layer. By using it as an intermediate layer, a solid solution can be formed *via* interdiffusion between adjacent layers, which further enhances the energy storage properties.^15,34^Fig. 12 shows SEM and EDS mapping images of the “sandwich” structure ceramic thick film composed of a 0.3NBT-0.1Sr-0.2Zr layer and an NBT interlayer (7 wt% glass, sintered at 900 °C). The thickness of the multilayer was ∼40 µm, the NBT layer ∼7 µm, and the single 0.3NBT-0.1Sr-0.2Zr layer ∼16 µm. According to the EDS analysis, interdiffusion occurred between the 0.3NBT-0.1Sr-0.2Zr and NBT layers, resulting in an indistinct interface. The Ag electrode was flat and continuous, with a clear interface visible against the ceramic layer. Compared with the single-layer film, interdiffusion between the 0.3NBT-0.1Sr-0.2Zr layers and the NBT interlayer created a compositionally graded solid-solution region. Such interdiffusion was expected to alleviate local stress concentration and further disrupt the long-range ferroelectric order, thereby enhancing relaxor behavior and interfacial polarization. As a result, an improvement in both the overall dielectric constant and temperature stability was anticipated. In addition, the compositional gradient within the sandwich structure would hinder the propagation of electrical trees, promote a more continuous polarization response across the layers, and reduce charge accumulation at the interfaces. Consequently, this architecture was expected to achieve a superior energy storage properties.

**Fig. 13:**
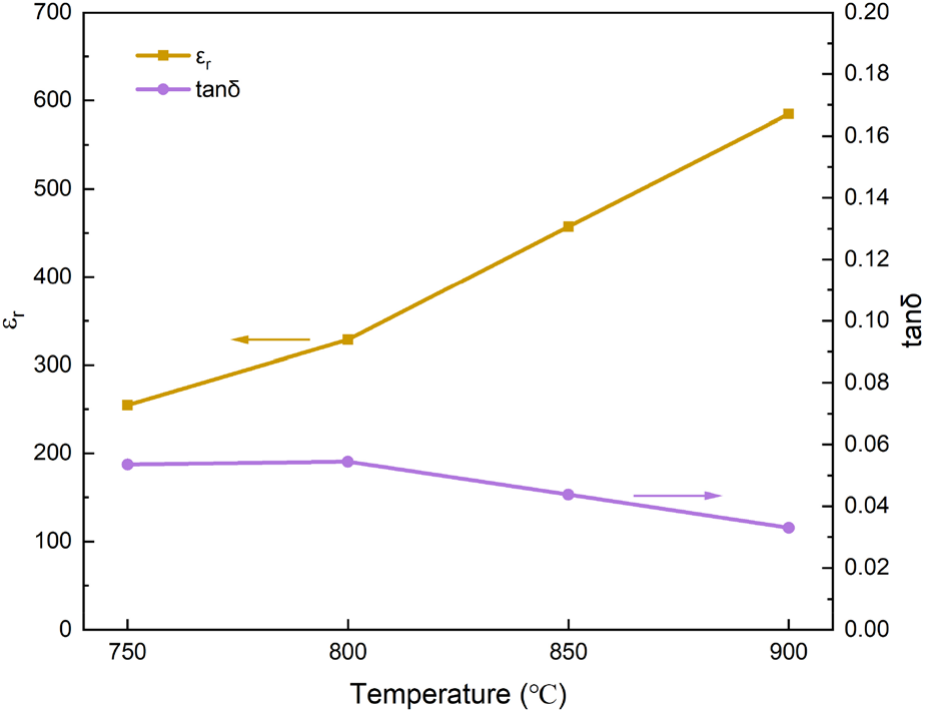
Dielectric properties curves of multilayer thick films (7 wt% glass) prepared at different sintering temperatures.

Fig. 13 shows the dielectric properties of the multilayer thick films prepared at different sintering temperatures (7 wt% glass). At the sintering temperature of 750 °C, the distribution between ceramic particles and between the liquid phase and ceramics was inhomogeneous. As a result, the dielectric constant remained relatively low, reaching a maximum of only 255. As the sintering temperature increased, the stronger sintering driving force led to more uniform particle distribution and increased formation of solid solutions *via* diffusion. This gradually increased the dielectric constant and reduced the dielectric loss. At 900 °C, the film achieved a dielectric constant of 585, the dielectric loss of 0.033.

**Fig. 14:**
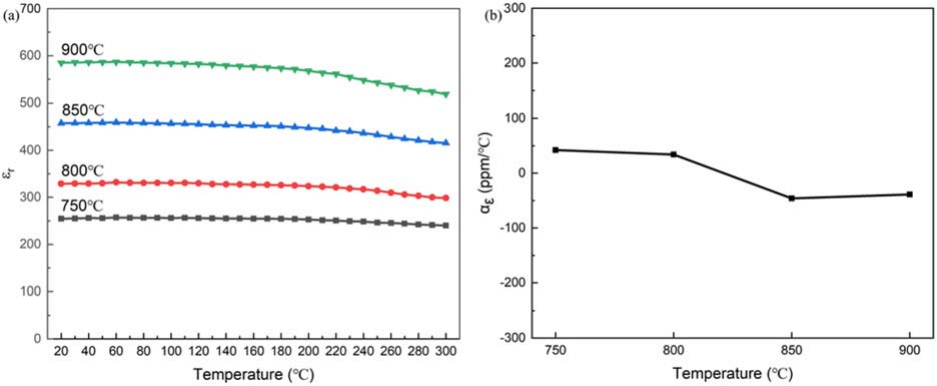
(a) Temperature-dependent dielectric constant curves (20–300 °C), (b) temperature coefficient of dielectric constant (20–120 °C) of multilayer thick films (7 wt% glass) prepared at different sintering temperatures.

Fig. 14 shows the temperature-dependent dielectric constant and corresponding temperature coefficients. Compared with the single-layer 0.3NBT-0.1Sr-0.2Zr ceramic films, the “sandwich” multilayer structure exhibited significantly enhanced dielectric properties, achieving a higher dielectric constant and a lower temperature coefficient of dielectric constant. At a sintering temperature of 750 °C, the temperature coefficient of dielectric constant was 42 ppm °C^−1^ (20–120 °C). As the sintering temperature increased, the dielectric constant gradually rose while the temperature coefficient remained stable. When the sintering temperature reached 900 °C, the dielectric constant reached its maximum, and the temperature coefficient of dielectric constant was only −39 ppm °C^−1^. Furthermore, it also exhibited excellent dielectric stability in the high-temperature region (>120 °C). NBT itself possesses a high dielectric constant and excellent relaxor behavior. Meanwhile, the interdiffusion between the NBT intermediate layer and the 0.3NBT-0.1Sr-0.2Zr layers formed interfacial solid-solution regions with compositional gradients, which enhanced the dielectric response.^39–41^ Furthermore, the “sandwich” structure effectively maintained stable dielectric response over a wider temperature range.^42–44^ These combined mechanisms enable the “sandwich” multilayer structure to achieve both significantly enhanced dielectric constant and excellent dielectric temperature stability.

**Fig. 15:**
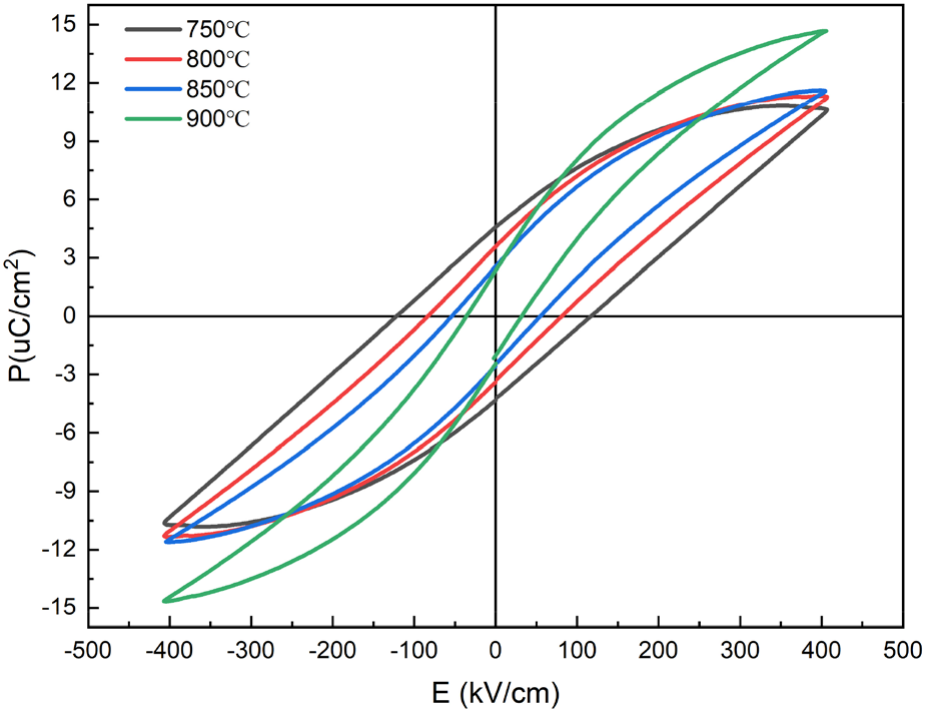
*P*–*E* loops of multilayer thick films (7 wt% glass) prepared at different sintering temperatures.

Fig. 15 shows the energy storage properties of the multilayer thick films prepared at different sintering temperatures. At the sintering temperature of 750 °C, ferroelectric domains were not fully developed and there were numerous structural defects. This resulted in an energy storage density of only 0.75 J cm^−3^, a high remanent polarization of 4.57 µC cm^−2^, and an energy storage efficiency of ∼38.79%. The increase in sintering temperature promoted the development of ferroelectric domains and structural densification, resulting in enhanced ferroelectric properties and reduced remanent polarization. At 900 °C, the film exhibited the breakdown strength of 405 kV cm^−1^, an energy storage density of 1.65 J cm^−3^, a remanent polarization of 2.34 µC cm^−2^, and an energy storage efficiency of 74.3%. Compared to the 0.3NBT-0.1Sr-0.2Zr ceramic film, the multilayer structure optimized the energy storage properties. The compositionally graded sandwich structure enhanced relaxor behavior and reduced charge accumulation at the interfaces.^18^ Furthermore, the structure introduced multiple interfaces that effectively inhibited the propagation of electrical trees, significantly enhancing the breakdown strength.^45^ In summary, the introduction of the “sandwich” structure systematically optimized the relaxor ferroelectricity and energy storage properties of the thick films through interlayer complementarity, interfacial diffusion, and multilayer blocking mechanisms.

In terms of screen printing technology, Zhang *et al.*^46–48^ used glass sintering aids to fabricate NBT-based ceramic films on Ag-Pb electrodes at a sintering temperature of 950 °C, obtaining a breakdown strength of 550–600 kV cm^−1^ and an energy storage density of 2.0–2.7 J cm^−3^. However, the energy storage efficiency was only ∼45%. For other preparation methods of BTO-based thick films, Yan *et al.*^49^ employed a film scraping process, achieving a breakdown strength of 350 kV cm^−1^ and an energy storage density of 1.92 J cm^−3^ at a sintering temperature of 1155 °C. Yang *et al.*^50^ used a tape-casting process, obtaining a breakdown strength of 410 kV cm^−1^ and an energy storage density of 2.35 J cm^−3^ at a sintering temperature of 1270 °C. Compared with the above works, this study exhibits good breakdown strength, energy storage density and excellent temperature stability, as well as lower sintering temperature and simpler preparation process.

## 4 Conclusions

In this study, a ZnO-B_2_O_3_-Bi_2_O_3_ lead-free glass is demonstrated to be an effective multifunctional sintering aid for screen-printed BTO-based relaxor ferroelectric thick films. Beyond lowering the sintering temperature to 900 °C, the glass simultaneously promotes densification and creates a microstructure in which an insulating glass shell isolates highly polar clusters. This design strategy provides a general route to simultaneously enhance breakdown strength and energy storage efficiency, which is a long-standing challenge in thick-film dielectric capacitors *via* screen printing. Furthermore, by introducing an NBT intermediate layer, a compositionally graded sandwich structure is constructed. The interdiffusion between layers led to a continuous polarization response and reduced charge accumulation at interfaces, achieving a synergistic enhancement of energy storage density and temperature stability. The optimal properties of the multilayer ceramic thick film are as follows: a dielectric constant of 585, a temperature coefficient of dielectric constant of −39 ppm °C^−1^ (20–120 °C), a breakdown strength of 405 kV cm^−1^, an energy storage density of 1.65 J cm^−3^, and an energy storage efficiency of 74.3%. The use of environmentally benign glass additives and the scalable screen-printing process align with the growing demand for green, low-cost, and high-power energy storage devices. The structure design may inspire future optimization of multilayer ceramic capacitors for pulsed power and automotive electronics applications.

## Author contributions

Conceptualization, H. Z. and W. M.; methodology, H. Z., Z. Z. and N. Y.; validation, Z. Z.; formal analysis, J. H. and W. M.; data curation, H. Z.; resources, W. M.; supervision, H. Z.; writing – original draft, H. Z.; writing – review and editing, W. M. All authors have read and agreed to the published version of the manuscript.

## Conflicts of interest

There are no conflicts to declare.

## Supplementary Material

RA-016-D6RA02102F-s001

RA-016-D6RA02102F-s002

## Data Availability

The original contributions presented in this study are included in the article and supplementary information file (SI). Further inquiries can be directed to the corresponding author. Supplementary information is available. See DOI: https://doi.org/10.1039/d6ra02102f.
