# Supplementary material for: Low-temperature fabrication of BTO-based relaxor ferroelectric thick films with multi-layered architecture
Source: RSC Adv. 2026 May 18;16(29):26238–48. doi: 10.1039/d6ra02102f (PMC13185808; doi:10.1039/d6ra02102f)
Supplement: RA-016-D6RA02102F-s001 [file RA-016-D6RA02102F-s001.zip › Data availability/schematic diagram.pptx]

## Slide 1
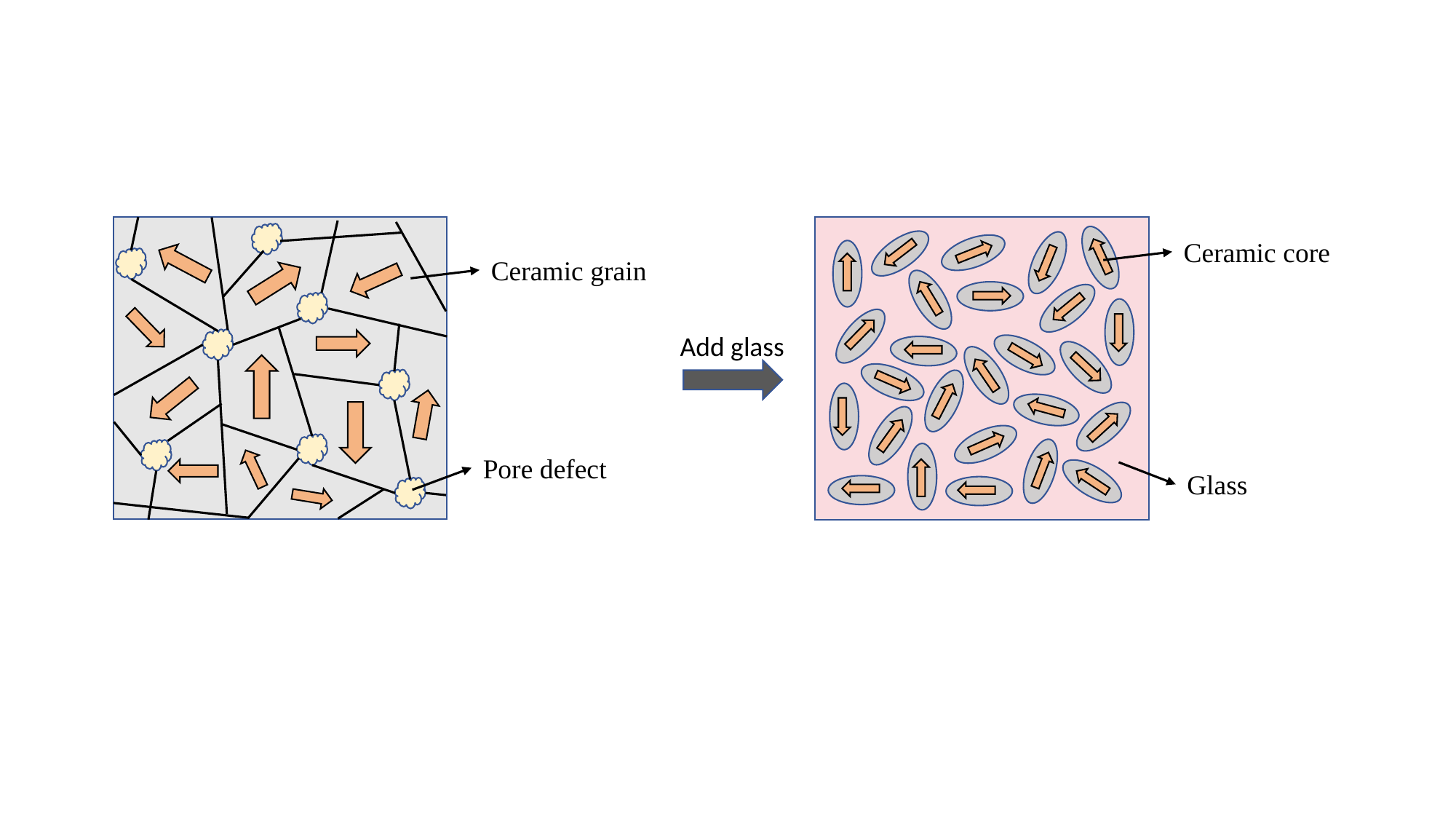

Ceramic grain
Pore defect
Ceramic core
Glass
Add glass

## Slide 2
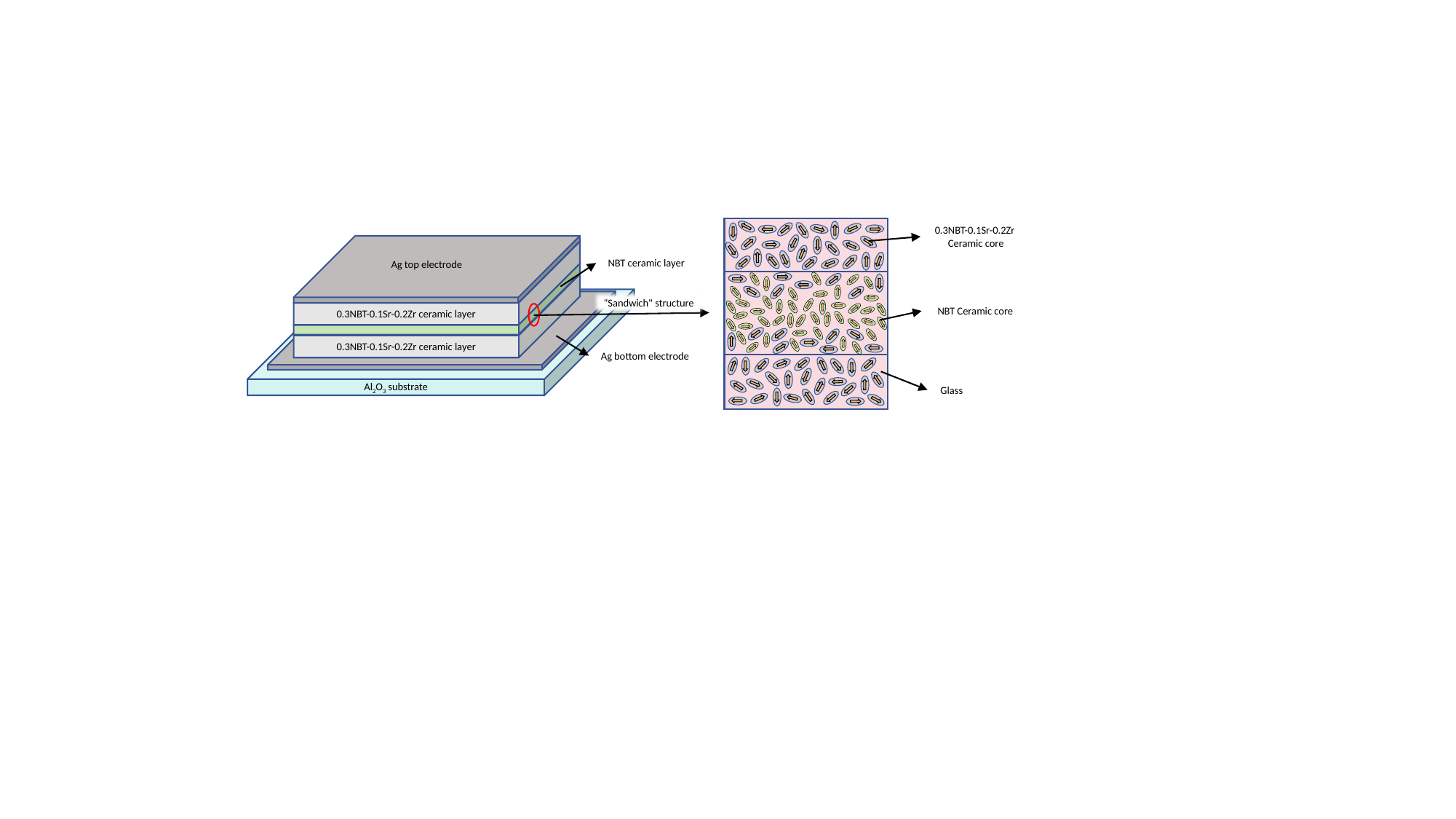

0.3NBT-0.1Sr-0.2Zr
Ceramic core
NBT Ceramic core
Glass
0.3NBT-0.1Sr-0.2Zr ceramic layer
NBT ceramic layer
Ag top electrode
0.3NBT-0.1Sr-0.2Zr ceramic layer
Al2O3 substrate
Ag bottom electrode
“Sandwich" structure
