# Supplementary figures and images for: Low-temperature fabrication of BTO-based relaxor ferroelectric thick films with multi-layered architecture
Source: RSC Adv. 2026 May 18;16(29):26238–48. doi: 10.1039/d6ra02102f (PMC13185808; doi:10.1039/d6ra02102f)

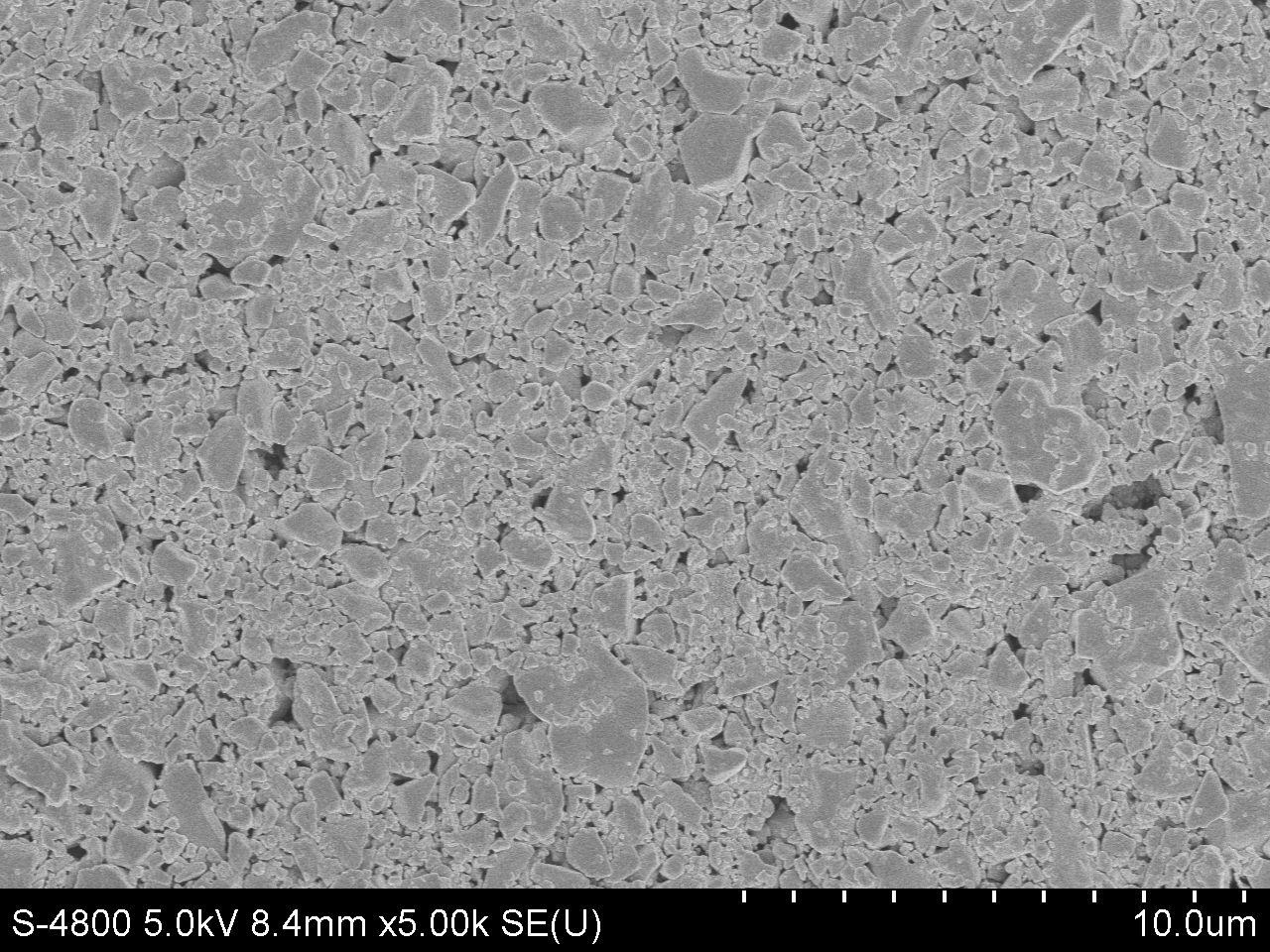

Supplement: RA-016-D6RA02102F-s001 [file RA-016-D6RA02102F-s001.zip › Data availability/SEM-EDS/0.3-0.1-0.2-0%-800-5k.tif]

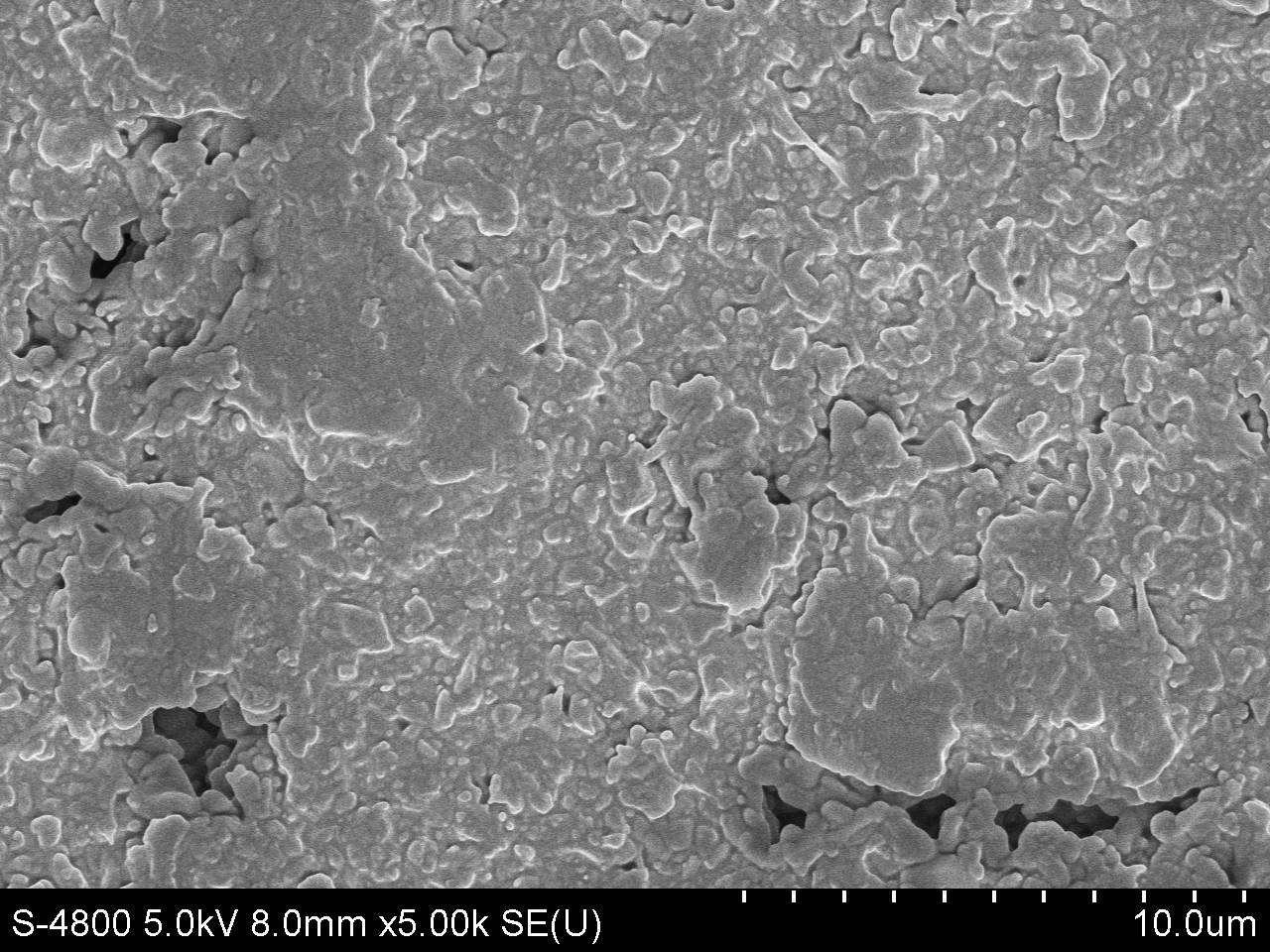

Supplement: RA-016-D6RA02102F-s001 [file RA-016-D6RA02102F-s001.zip › Data availability/SEM-EDS/0.3-0.1-0.2-3%-800-5k.tif]

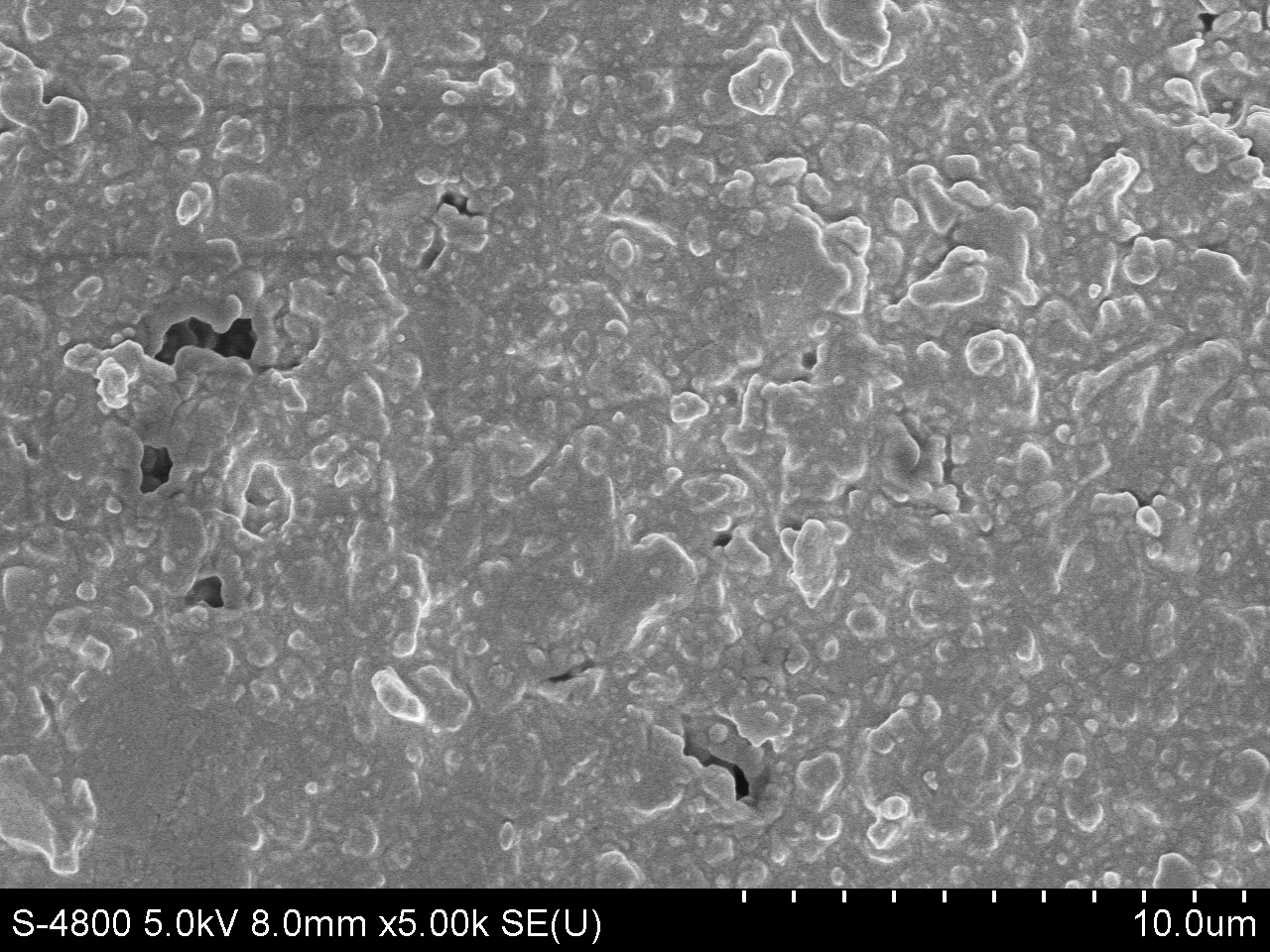

Supplement: RA-016-D6RA02102F-s001 [file RA-016-D6RA02102F-s001.zip › Data availability/SEM-EDS/0.3-0.1-0.2-5%-800-5k.tif]

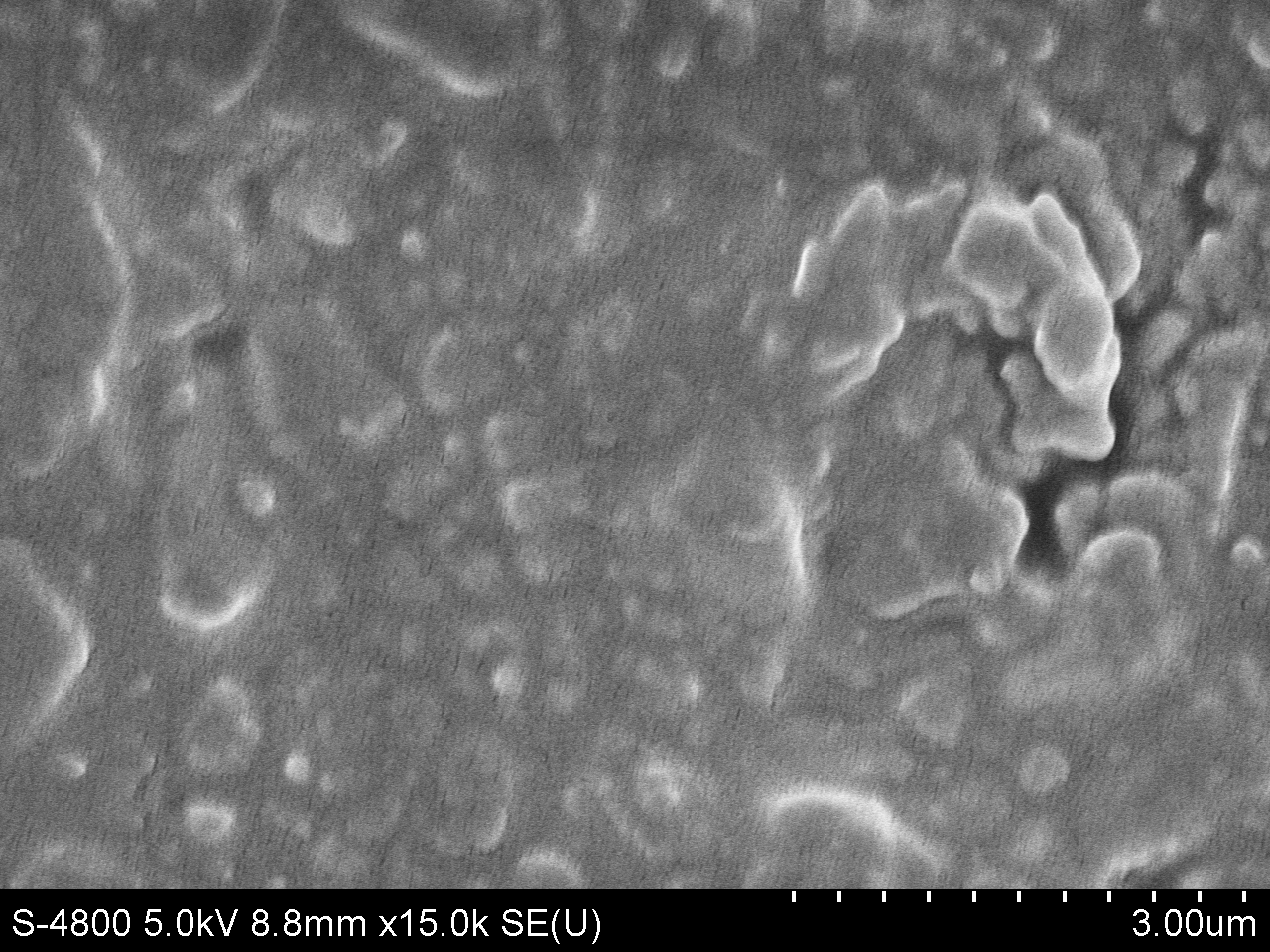

Supplement: RA-016-D6RA02102F-s001 [file RA-016-D6RA02102F-s001.zip › Data availability/SEM-EDS/0.3-0.1-0.2-7%-750-15k.tif]

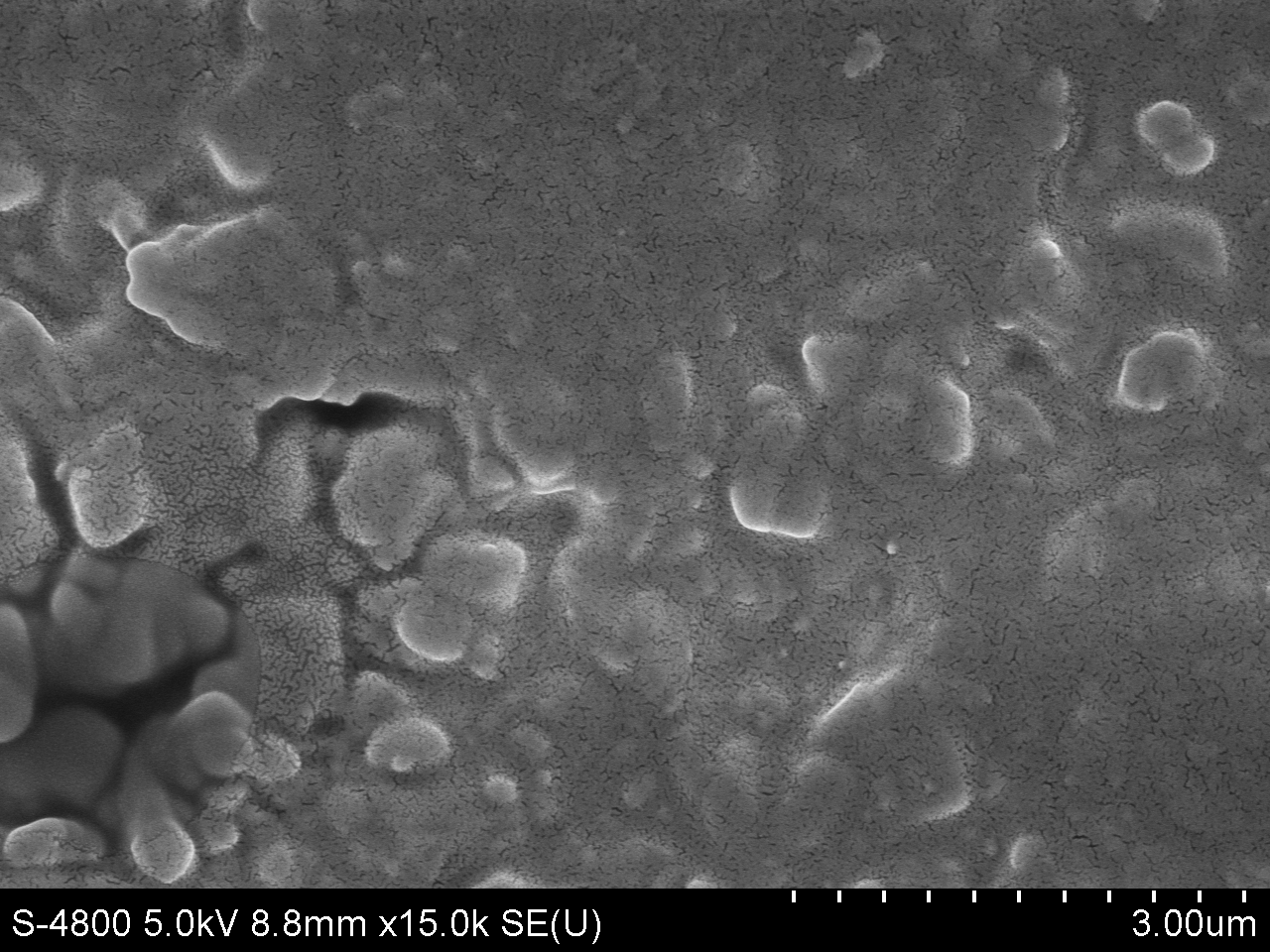

Supplement: RA-016-D6RA02102F-s001 [file RA-016-D6RA02102F-s001.zip › Data availability/SEM-EDS/0.3-0.1-0.2-7%-800-15k.tif]

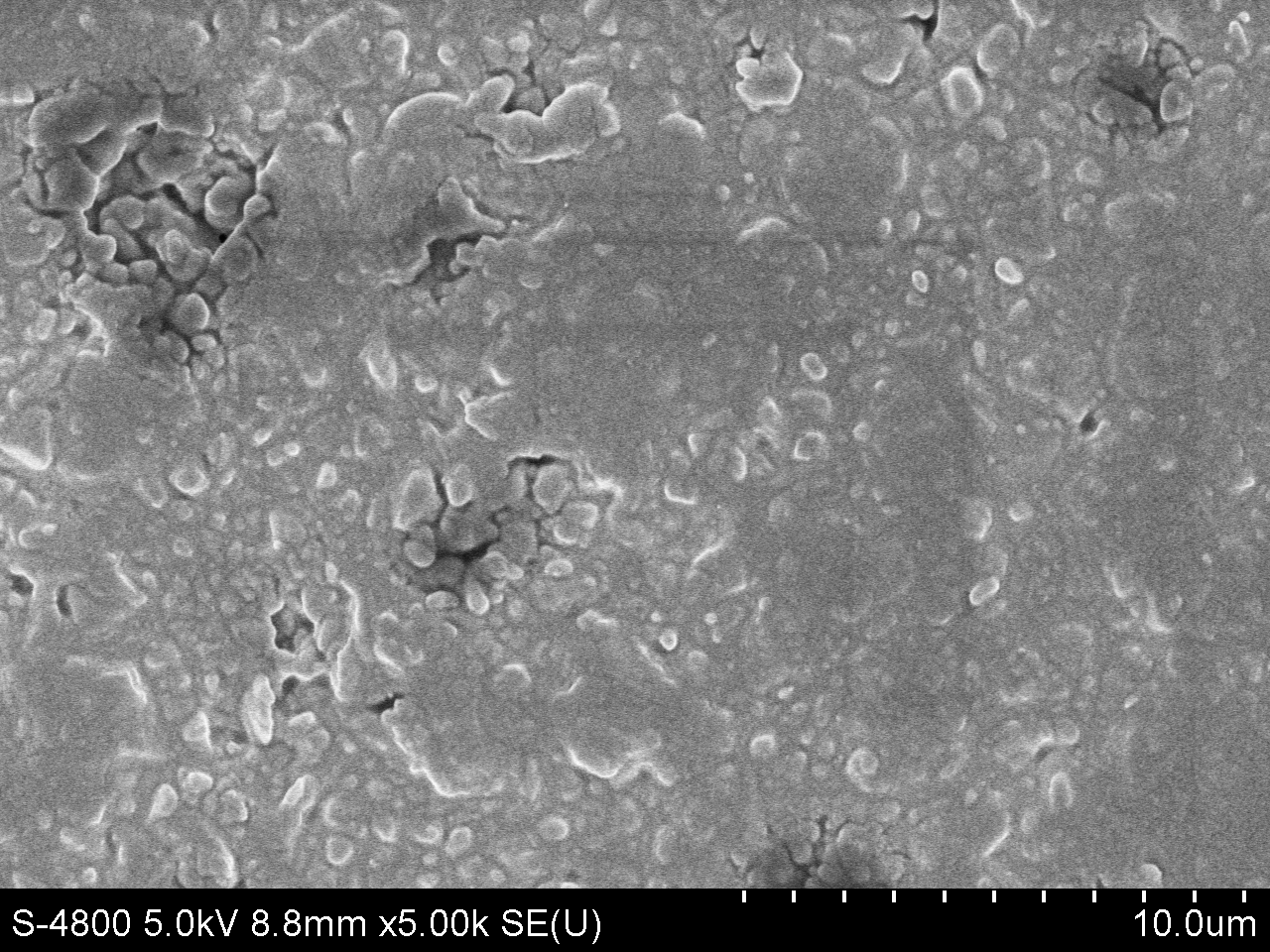

Supplement: RA-016-D6RA02102F-s001 [file RA-016-D6RA02102F-s001.zip › Data availability/SEM-EDS/0.3-0.1-0.2-7%-800-5k.tif]

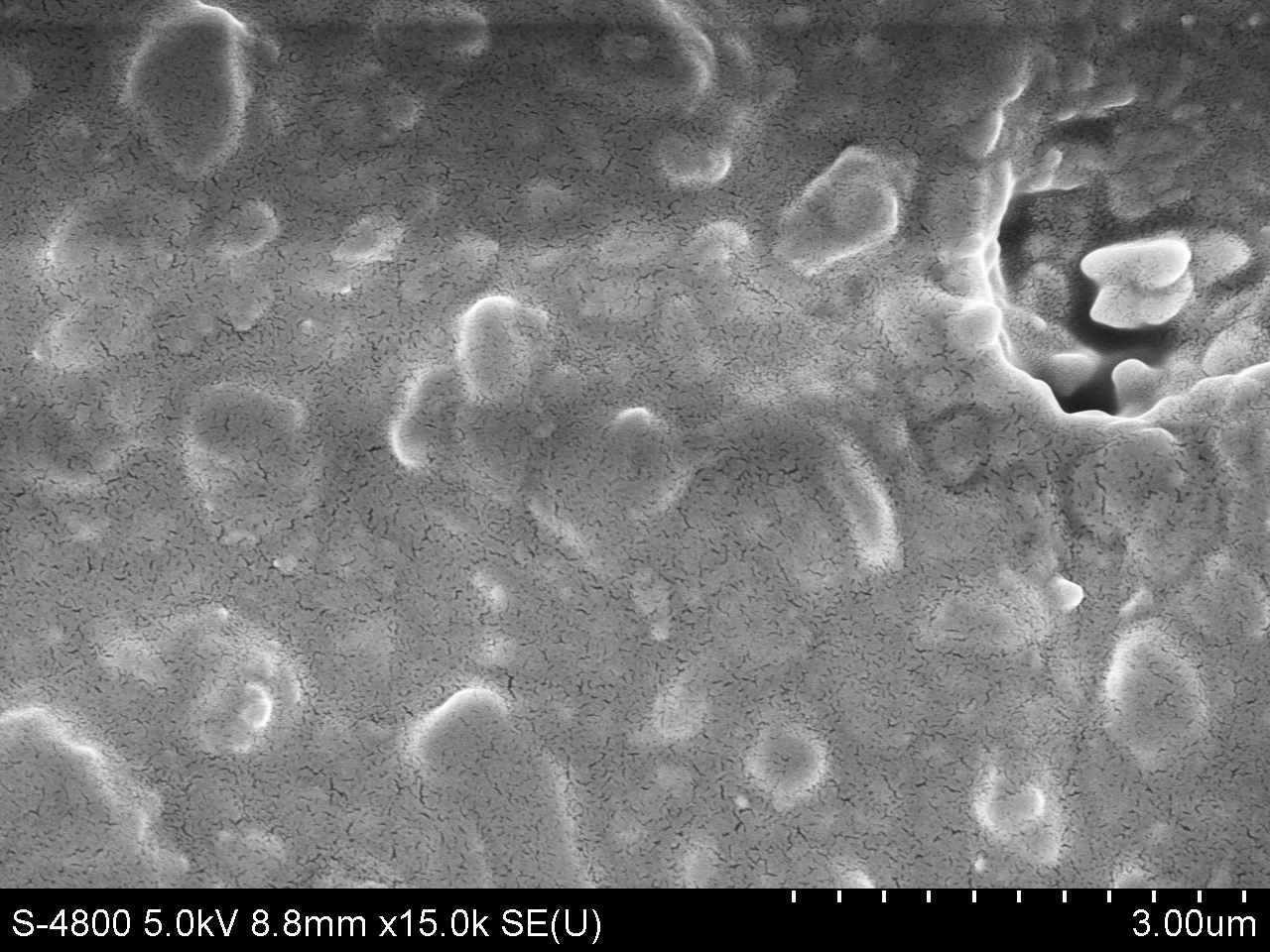

Supplement: RA-016-D6RA02102F-s001 [file RA-016-D6RA02102F-s001.zip › Data availability/SEM-EDS/0.3-0.1-0.2-7%-850-15k.tif]

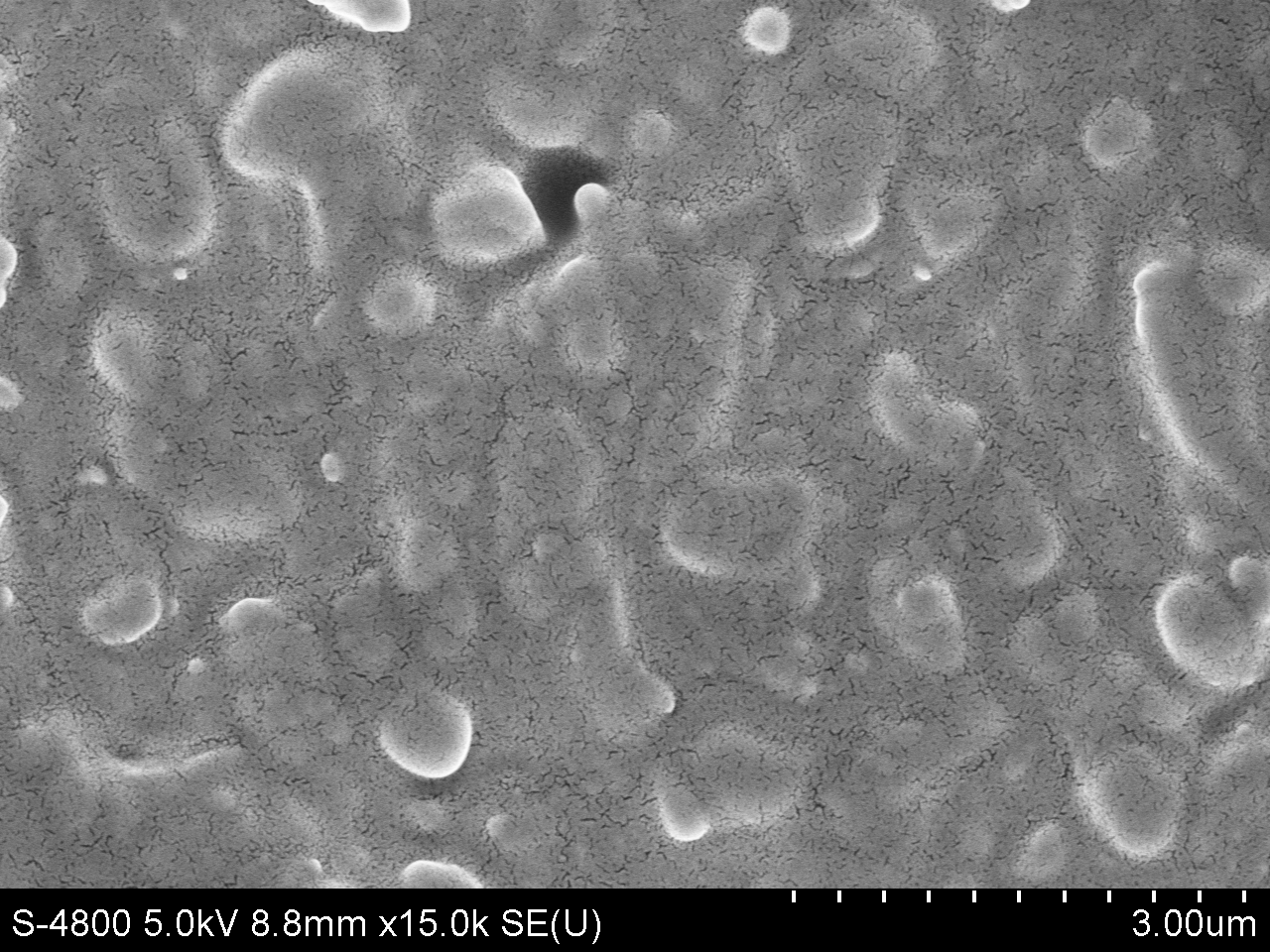

Supplement: RA-016-D6RA02102F-s001 [file RA-016-D6RA02102F-s001.zip › Data availability/SEM-EDS/0.3-0.1-0.2-7%-900-15k.tif]

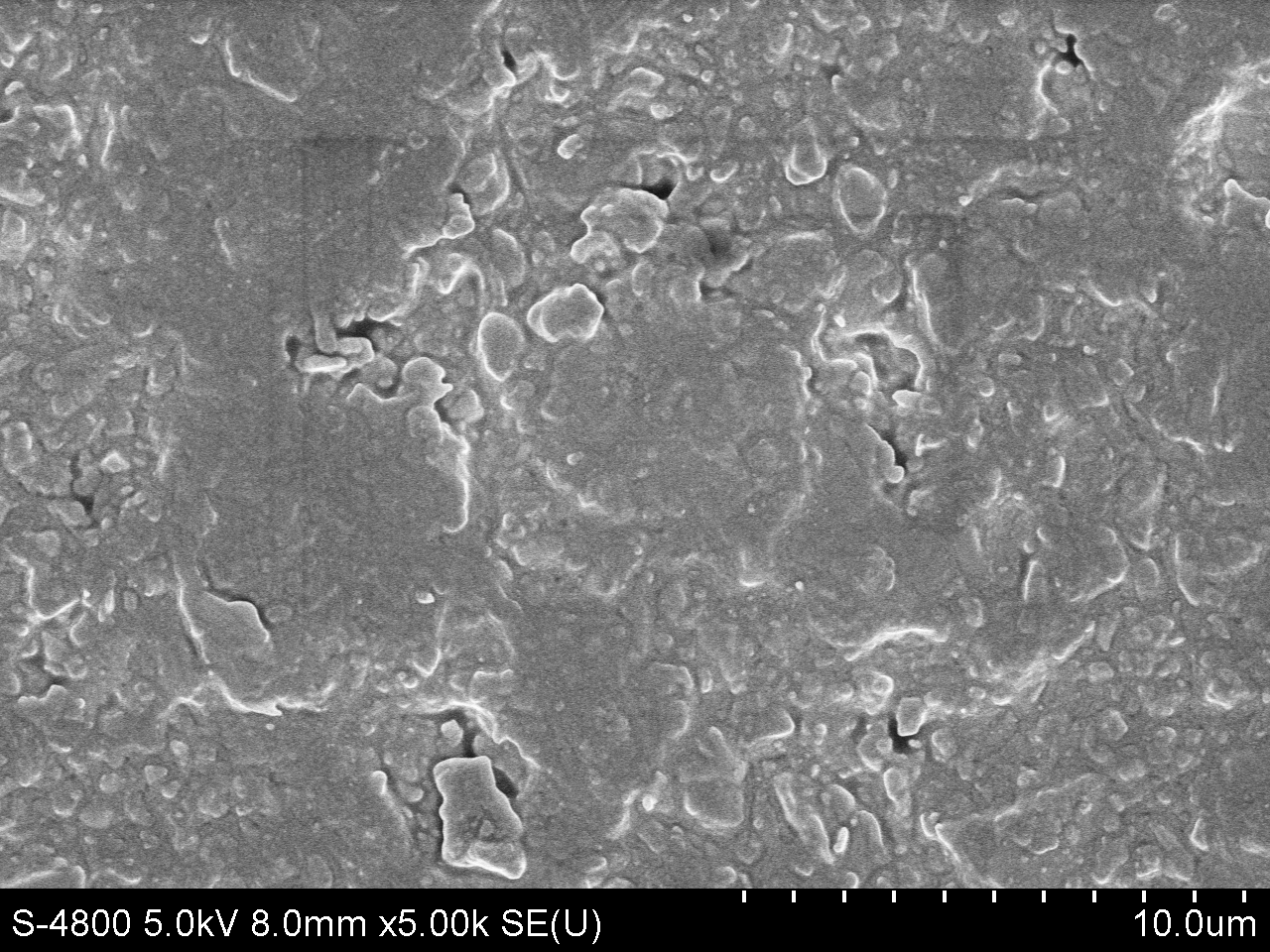

Supplement: RA-016-D6RA02102F-s001 [file RA-016-D6RA02102F-s001.zip › Data availability/SEM-EDS/0.3-0.1-0.2-9%-800-5k.tif]

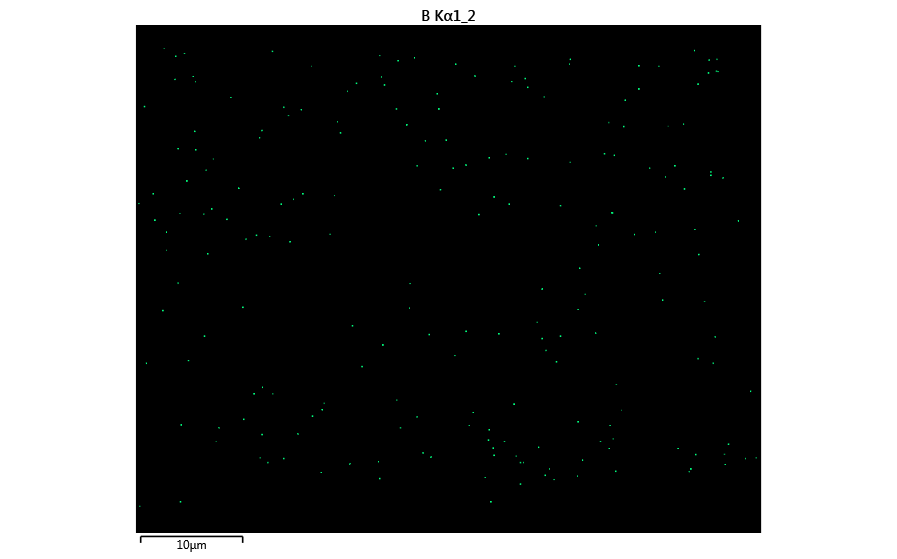


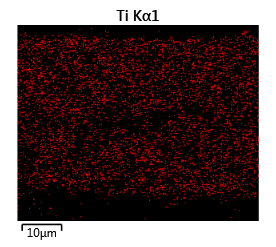

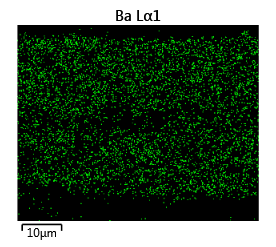

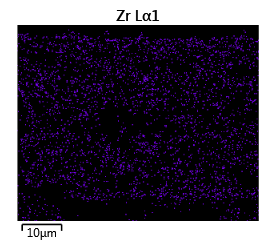

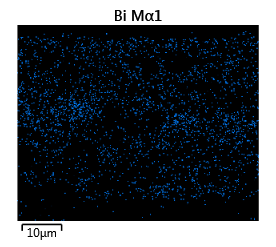

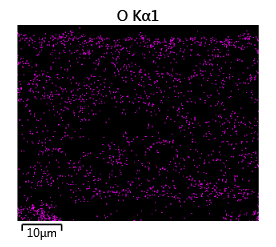

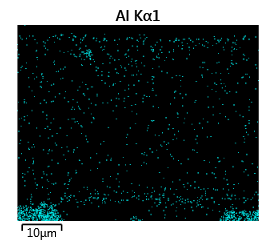

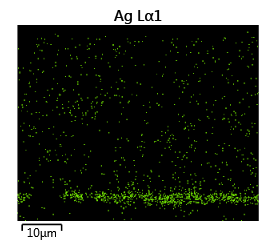

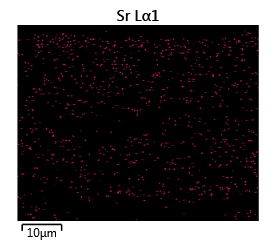

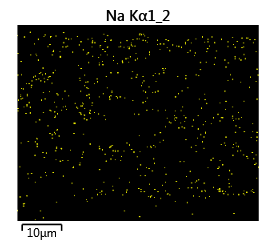

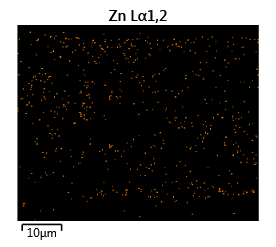

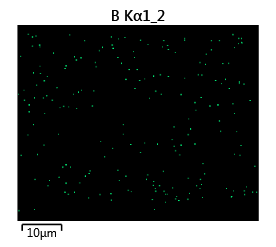

Supplement: RA-016-D6RA02102F-s001 [file RA-016-D6RA02102F-s001.zip › Data availability/SEM-EDS/multilayer film-EDS.docx]
