# Supplementary material for: Low-temperature fabrication of BTO-based relaxor ferroelectric thick films with multi-layered architecture
Source: RSC Adv. 2026 May 18;16(29):26238–48. doi: 10.1039/d6ra02102f (PMC13185808; doi:10.1039/d6ra02102f)
Supplement: RA-016-D6RA02102F-s002 [file RA-016-D6RA02102F-s002.pdf]

Supplementary Information (SI) for RSC Advances.
This journal is © The Royal Society of Chemistry 2026

Low-temperature fabrication of BTO-based relaxor ferroelectric thick films with multi-layered architecture

HongweiZhang[a], WeibingMa[a*], ZhuoZhang[a], NaiheYi[a] and JingNanHong[a]

[a]Key Laboratory of Advanced Ceramics and Machining Technology of Ministry of Education, Tianjin University, Tianjin 300072, China, maweibing@tju.edu.cn

The main raw materials used in this experiment are shown in Table S1.

Table S1 Raw materials used in the experiment

Name	Molecular Formula	Purity	Manufacturer Information
Barium Carbonate	$BaCO_3$	A.R.	Shanghai Aladdin Chemical Reagent Co., Ltd
Strontium Carbonate	$SrCO_3$	A.R.	Shanghai Aladdin Chemical Reagent Co., Ltd
Titanium Dioxide	TiO_2	A.R.	Shanghai Aladdin Chemical Reagent Co., Ltd
Zirconium Dioxide	ZrO_2	A.R.	Tianjin Kemio Chemical Reagent Co., Ltd
Sodium Carbonate	Na_2CO_3	A.R.	China National Pharmaceutical Group Chemical Reagent Co., Ltd
Bismuth Trioxide	Bi2O3	A.R.	Shanghai Aladdin Chemical Reagent Co., Ltd
Zinc Oxide	ZnO	A.R.	Shanghai Aladdin Chemical Reagent Co., Ltd
Boron Trioxide	B_2O_3	A.R.	Shanghai Aladdin Chemical Reagent Co., Ltd

The main equipment used in this experiment is listed in Table S2.

Table S2 Main equipment used in this experiment

Name	Model	Manufacturer Information
Precision Balance	BSA2245S-CW	Sadolis Scientific Instruments Co., Ltd
Planetary Ball Mill	ND8-2L	Nanjing Nanda Tianzun Electronics Co., Ltd
Heating Magnetic Stirrer	DF-101S	Tianjin Kono Instrument Equipment Co., Ltd
Blast Drying Oven	YLE-2000	Tianjin Shunnuo Instrument Technology Co., Ltd
Box Furnace	KF 1200	Nanjing Boyuntang Instrument Technology Co., Ltd

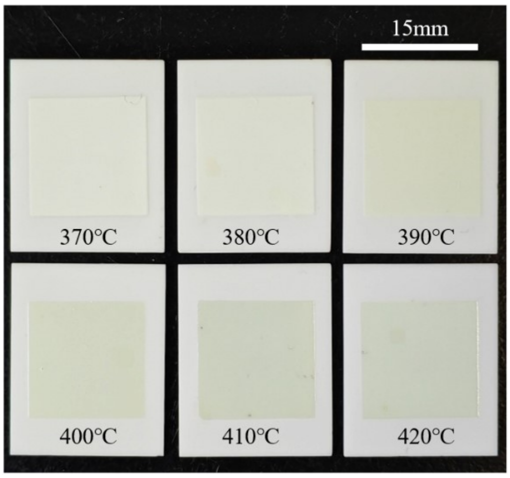

Figure S1 Pictures of the melting state of glass after different sintering temperatures

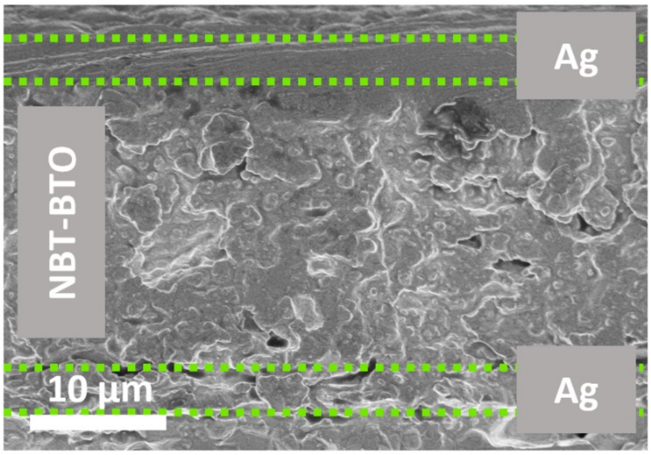

Figure S2 Cross-sectional SEM images of 0.3NBT-0.1Sr-0.2Zr thick film with 7 wt.% glass sintered at 900 ℃

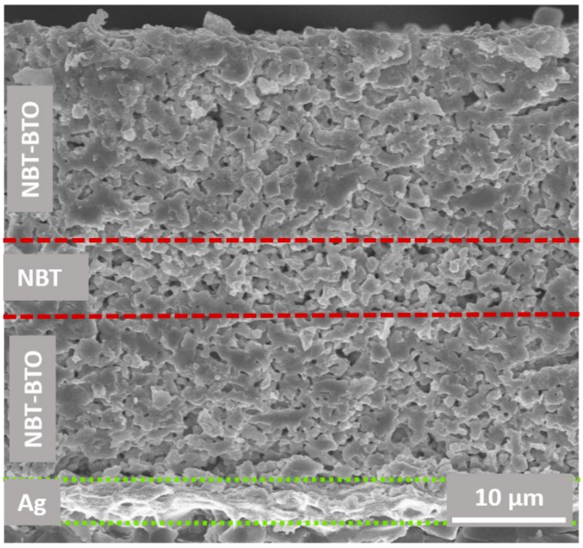

Figure S3 Cross-sectional SEM images of sandwiched multilayer ceramic thick film with 7 wt.% glass sintered at 900 ℃
